# Prioritizing Genetic Contributors to Cortical Alterations in 22q11.2 Deletion Syndrome Using Imaging Transcriptomics

**DOI:** 10.1093/cercor/bhab008

**Published:** 2021-02-26

**Authors:** Jennifer K Forsyth, Eva Mennigen, Amy Lin, Daqiang Sun, Ariana Vajdi, Leila Kushan-Wells, Christopher R K Ching, Julio E Villalon-Reina, Paul M Thompson, 22q11.2 ENIGMA Consortium, Rachel K Jonas, Laura Pacheco-Hansen, Geor Bakker, Therese van Amelsvoort, Kevin M Antshel, Wanda Fremont, Wendy R Kates, Linda E Campbell, Kathryn L McCabe, Michael C Craig, Eileen Daly, Maria Gudbrandsen, Clodagh M Murphy, Declan G Murphy, Kieran C Murphy, Ania Fiksinski, Sanne Koops, Jacob Vorstman, T Blaine Crowley, Beverly S Emanuel, Raquel E Gur, Donna M McDonald-McGinn, David R Roalf, Kosha Ruparel, J Eric Schmitt, Elaine H Zackai, Courtney A Durdle, Naomi J Goodrich-Hunsaker, Tony J Simon, Anne S Bassett, Nancy J Butcher, Eva W C Chow, Fidel Vila-Rodriguez, Adam Cunningham, Joanne L Doherty, David E Linden, Hayley Moss, Michael J Owen, Marianne van den Bree, Nicolas A Crossley, Gabriela M Repetto, Carrie E Bearden

**Affiliations:** Department of Psychiatry and Biobehavioral Sciences, University of California at Los Angeles, Los Angeles, CA 90095, USA; Semel Institute for Neuroscience and Human Behavior, University of California at Los Angeles, Los Angeles, CA 90024, USA; Department of Psychiatry and Biobehavioral Sciences, University of California at Los Angeles, Los Angeles, CA 90095, USA; Semel Institute for Neuroscience and Human Behavior, University of California at Los Angeles, Los Angeles, CA 90024, USA; Department of Psychiatry and Psychotherapy, University Hospital Carl Gustav Carus, Technische Universität Dresden, Dresden 01307, Germany; Department of Psychiatry and Biobehavioral Sciences, University of California at Los Angeles, Los Angeles, CA 90095, USA; Semel Institute for Neuroscience and Human Behavior, University of California at Los Angeles, Los Angeles, CA 90024, USA; Interdepartmental Neuroscience Program, University of California at Los Angeles, Los Angeles, CA 90095, USA; Department of Psychiatry and Biobehavioral Sciences, University of California at Los Angeles, Los Angeles, CA 90095, USA; Semel Institute for Neuroscience and Human Behavior, University of California at Los Angeles, Los Angeles, CA 90024, USA; Veterans Affairs Greater Los Angeles Healthcare System, Los Angeles, CA 90073, USA; Department of Psychiatry and Biobehavioral Sciences, University of California at Los Angeles, Los Angeles, CA 90095, USA; Semel Institute for Neuroscience and Human Behavior, University of California at Los Angeles, Los Angeles, CA 90024, USA; Department of Psychiatry and Biobehavioral Sciences, University of California at Los Angeles, Los Angeles, CA 90095, USA; Semel Institute for Neuroscience and Human Behavior, University of California at Los Angeles, Los Angeles, CA 90024, USA; Imaging Genetics Center, Mark and Mary Stevens Neuroimaging and Informatics Institute, Keck School of Medicine, University of Southern California, Los Angeles, CA 90033, USA; Imaging Genetics Center, Mark and Mary Stevens Neuroimaging and Informatics Institute, Keck School of Medicine, University of Southern California, Los Angeles, CA 90033, USA; Imaging Genetics Center, Mark and Mary Stevens Neuroimaging and Informatics Institute, Keck School of Medicine, University of Southern California, Los Angeles, CA 90033, USA; Department of Psychiatry and Biobehavioral Sciences, University of California at Los Angeles, Los Angeles, CA 90095, USA; Semel Institute for Neuroscience and Human Behavior, University of California at Los Angeles, Los Angeles, CA 90024, USA; Brain Research Institute, University of California at Los Angeles, Los Angeles, CA 90095, USA; Department of Psychology, University of California at Los Angeles, Los Angeles, CA 90095, USA

**Keywords:** copy number variant, cortical thickness, *DGCR8*, gene expression, surface area

## Abstract

22q11.2 deletion syndrome (22q11DS) results from a hemizygous deletion that typically spans 46 protein-coding genes and is associated with widespread alterations in brain morphology. The specific genetic mechanisms underlying these alterations remain unclear. In the 22q11.2 ENIGMA Working Group, we characterized cortical alterations in individuals with 22q11DS (*n* = 232) versus healthy individuals (*n* = 290) and conducted spatial convergence analyses using gene expression data from the Allen Human Brain Atlas to prioritize individual genes that may contribute to altered surface area (SA) and cortical thickness (CT) in 22q11DS. Total SA was reduced in 22q11DS (Z-score deviance = −1.04), with prominent reductions in midline posterior and lateral association regions. Mean CT was thicker in 22q11DS (Z-score deviance = +0.64), with focal thinning in a subset of regions. Regional expression of *DGCR8* was robustly associated with regional severity of SA deviance in 22q11DS; *AIFM3* was also associated with SA deviance. Conversely, *P2RX6* was associated with CT deviance. Exploratory analysis of gene targets of microRNAs previously identified as down-regulated due to *DGCR8* deficiency suggested that *DGCR8* haploinsufficiency may contribute to altered corticogenesis in 22q11DS by disrupting cell cycle modulation. These findings demonstrate the utility of combining neuroanatomic and transcriptomic datasets to derive molecular insights into complex, multigene copy number variants.

## Introduction

22q11.2 deletion syndrome (22q11DS) arises from the deletion of a segment of chromosome 22 due to misalignment of low copy repeats (LCR) during nonallelic homologous recombination. It occurs in approximately 1 in 3000–4000 births and spans a ~2.6 megabase (Mb) region that results in the hemizygous deletion of 46 protein-coding genes in 85–90% of patients (Guna et al. 2015), with ~10–15% of 22q11DS patients carrying a smaller, nested deletion (McDonald-McGinn et al. 2015). 22q11DS is associated with a broad phenotype that includes heart anomalies, immune dysfunction, and high rates of neuropsychiatric and neurodevelopmental disorders such as schizophrenia, intellectual disability, and autism spectrum disorder (ASD; Jonas et al. 2014; Schneider et al. 2014). Alterations in brain structure and function are thought to contribute to the psychiatric and developmental phenotypes frequently observed in the disorder.

Indeed, it is now established that 22q11DS is associated with widespread alterations in brain morphology. Early magnetic resonance imaging (MRI) studies reported whole brain volumetric reductions in 22q11DS, with greater reductions in midline regions, as well as in posterior relative to anterior regions (Tan et al. 2009; Karayiorgou et al. 2010). However, as cortical gray matter volume reflects the product of cortical surface area (SA; i.e., area covered by the cortex) and cortical thickness (CT; i.e., thickness of the 6 neocortical layers), which appear to be determined through relatively independent genetic and neurodevelopmental mechanisms (Panizzon et al. 2009; Winkler et al. 2010; Chen et al. 2013; Grasby et al. 2020, but see also Schmitt et al. 2018), recent studies have examined these morphometric characteristics separately. Thus, brain volume reductions in 22q11DS were recently found to be driven by widespread reductions in SA (Sun et al. 2019). Conversely, CT tends to be increased in 22q11DS, with focal thinning in only a minority of regions. Overall, the magnitude of SA alterations in 22q11DS is roughly 2-fold the magnitude of CT alterations (Sun et al. 2020). Importantly, while 22q11.2 deletions yield reduced expression of the majority of genes within the locus (Stark et al. 2008; Jalbrzikowski et al. 2015; Lin et al. 2016; Gordon et al. 2019), all genes within the locus are not expected to contribute equally to brain phenotypes in the disorder (Motahari et al. 2019). As neuroanatomic abnormalities are associated with a range of neuropsychiatric and developmental phenotypes, clarifying the individual genes underlying these abnormalities may provide insight into molecular mechanisms that contribute to broader psychiatric and developmental phenotypes in 22q11DS.

Leveraging comprehensive maps of gene expression in the human brain offers one promising approach to identify molecular mechanisms underlying neuroanatomic deviations in 22q11DS. Recent studies have used the Allen Human Brain Atlas (AHBA), a transcriptomic dataset quantifying the expression of over 20 000 genes across postmortem brain tissue from six psychiatrically healthy individuals, to elucidate mechanisms underlying cellular and neural circuit variation in healthy individuals and in populations with neuropsychiatric and neurodegenerative disorders (Fornito et al. 2019). By examining the spatial convergence between brain phenotypes and gene expression patterns, recent studies found that brain regions that are closer in physical proximity (Hawrylycz et al. 2012) or have functionally correlated activity (Richiardi et al. 2015) share more similar transcriptomic expression patterns. Similarly, a prominent rostro-caudal gradient of gene expression has been found across the cortex (Bernard et al. 2012; Hawrylycz et al. 2012; Miller et al. 2014), which is thought to reflect the rostro-caudal gradient of neurogenesis and cell composition in which posterior brain regions have a higher density of neurons that are smaller in size, while anterior regions tend to have a lower density of neurons that are larger in size and spine density (Cahalane et al. 2012; Charvet et al. 2015; Fornito et al. 2019). In clinical populations, the pattern of structural dysconnectivity in schizophrenia patients was spatially correlated with the expression of 43 genes previously implicated in schizophrenia by genome-wide association (Romme et al. 2017); regional expression of the Parkinson’s risk gene, *MAPT*, was spatially correlated with the topography of connectivity differences in patients with Parkinson’s disease (Rittman et al. 2016); and regional expression of transcriptionally down-regulated genes in postmortem cortex of ASD patients was associated with severity of CT deviation in ASD (Romero-Garcia 2019). Spatial convergence analyses were also recently applied in a 16p11.2 deletion mouse model to identify genes within the locus that may be causally related to structural brain changes associated with the copy number variant (CNV; Kumar et al. 2018). Thus, prior studies of neurodevelopmental, neuropsychiatric, and neurodegenerative populations suggest that identifying genes with expression patterns that are spatially correlated with neuroimaging phenotypes can offer a useful strategy to elucidate genetic drivers of altered brain structure and function.

Here, as part of the 22q11.2 Enhancing Neuroimaging Genetics through Meta-Analysis (ENIGMA) Working Group (Thompson et al. 2020), we therefore integrated neuroanatomic data from a large multicenter cohort of 22q11DS individuals with molecularly confirmed deletions spanning the full LCR A-D region and transcriptomic data from the AHBA. By characterizing the spatial convergence between regional expression of each individual 22q11.2 gene and the severity of morphometric alterations within patients, we sought to systematically prioritize individual genes within the 22q11.2 locus that may be causally related to these alterations and elucidate potential underlying molecular mechanisms.

## Materials and Methods

### Structural MRI Data

Structural MRI (sMRI) data from 386 22q11DS patients and 315 typical developing controls analyzed in a previously published study from the 22q11DS ENIGMA working group (Sun et al. 2020) were used to derive measures of SA and CT deviance in 22q11DS for the current study. Briefly, in the original study, data were pooled across nine study sites with patient and control data. FreeSurfer image processing software (version 5.3.0; http://surfer.nmr.mgh.harvard.edu) was used to process 1 mm^3^ *T*_1_-weighted structural images acquired with an MPRAGE sequence. Quality control was implemented using validated and standardized processing pipelines developed for the ENIGMA consortium (Thompson et al. 2014, 2017; http://enigma.ini.usc.edu/protocols/imaging-protocols). Total intracranial volume (ICV) and SA and CT measures for 68 cortical regions (34 per hemisphere) were calculated based on the Desikan-Killiany atlas. Group effects in this multisite 22q11DS cohort were previously found to be highly consistent across sites (Sun et al. 2020).

The purpose of the current analysis was to examine whether the regional expression patterns of individual genes in the 22q11.2 locus are associated with regional severity of SA or CT deviance in 22q11DS patients. Consequently, our analyses focused on a homogeneous sample of 22q11DS patients with the full ~2.6 Mb A-D deletion and the typical expression patterns (i.e., in healthy individuals) of the corresponding genes within this region, based on the AHBA. 22q11.2 deletion breakpoints for each patient were determined using multiplex ligation-dependent probe amplification (MLPA; Vorstman et al. 2006), which is a polymerase chain reaction (PCR)-based assay that can detect copy number deletions and duplications for up to 50 DNA probe sequences in one reaction. Due to its low cost, high sensitivity and specificity, and medium throughput, it is considered a gold standard method for CNV genotyping in humans (Kerkhof et al. 2017). MLPA for the current study was completed using the SALSA MLPA Probemix P250-B2 DiGeorge kit from MRC-Holland, which includes 29 probe sequences within the 22q11.2 locus to discern between common 22q11.2 deletion subtypes. Sites or scanners with no 22q11DS patients with a confirmed A-D deletion or corresponding healthy control data were excluded from the current analyses, leaving data from 232 22q11DS patients with confirmed A-D deletions and 290 controls collected on 10 scanners across 8 sites for analysis. Regional SA measures were adjusted for effects of age, sex, and site/scanner; different scanners were treated as independent “sites.” Regional CT measures were additionally adjusted for age^2^, based on significant nonlinear effects of age previously found in most ROIs for CT (Sun et al. 2020).

Subject consent at each site was obtained according to the Declaration of Helsinki, and study protocols were approved by ethical committees at each institution. Detailed information on the sample recruitment procedures, image acquisition parameters, and data processing are published elsewhere (Sun et al. 2020).

### Gene Expression Data

AHBA transcriptomic data for 20 737 largely protein-coding genes, registered to the Desikan-Killiany cortical atlas for integration with FreeSurfer-based analyses, were obtained from https://figshare.com/articles/A_FreeSurfer_view_of_the_cortical_transcriptome_generated_from_the_Allen_Human_Brain_Atlas/1439749 (French and Paus 2015). The original AHBA assayed the expression of 58 692 probes at a high spatial resolution, using custom Agilent arrays in 3702 brain samples derived from six healthy adults with no known neuropsychiatric or neuropathological history (Hawrylycz et al. 2012). In the French and Paus (2015) atlas, expression values from multiple probes for a given gene were first averaged to yield one expression value per gene per tissue sample. Each cortical brain tissue sample was then mapped to the nearest Desikan-Killiany cortical region based on its Montreal Neurological Institute coordinates, and for each individual brain, the median expression value across tissue samples mapping to a given Desikan-Killiany region was calculated for each gene. The median expression level across the six brains, per region, was then calculated for each of the 20 737 genes (see French and Paus 2015 for details). Because expression levels expression levels were measured from left hemisphere regions in all six brains, but only in two brains for right hemisphere regions, all region-based analyses used only left hemisphere regions.

To define protein-coding genes within the 22q11.2 locus, coordinates for the 22q11.2 locus were obtained from genome-wide studies of CNVs associated with schizophrenia (Marshall et al. 2017) and ASD (Sanders et al. 2015). Marshall et al. (2017) reported CNV borders in hg18; the UCSC LiftOver tool was used to convert them to hg19. As the 22q11.2 locus defined in these studies shared more than 90% overlap in basepairs (bp), the final 22q11.2 boundaries were defined as the union between those identified in these two studies (Forsyth et al. 2020). HGNC gene symbols for protein-coding genes within the locus were retrieved from Ensembl using the BioMart package in R (Durinck et al. 2009). Genes with mean log_2_ expression levels > 5, averaged across brain regions, were considered brain expressed, leaving 28 brain-expressed 22q11.2 protein-coding genes for investigation. The consistency of the expression of each 22q11.2 gene across the six donors, as defined by French and Paus (2015), is reproduced in the Supplementary Information.

## Statistical Analyses

### Demographic Characteristics

Group differences in age and sex were assessed with a univariate ANOVA and a chi-squared test, respectively.

### Neuroanatomic Group Differences

To characterize neuroanatomic alterations in the 22q11DS patients included in the present analyses, global and regional SA and CT metrics were first adjusted for age, sex, and scanner effects by conducting linear models with each neuroanatomic metric set as the dependent variable and with age, sex, and scanner set as independent variables, additionally including age^2^ for CT measures (Sun et al. 2020). Residualized values for each subject and neuroanatomic metric were retained from the linear models. Group differences in covariate-adjusted total SA and in mean CT were then examined using general linear models with total SA or mean CT as the dependent variable and group as the independent variable. Group differences in covariate-adjusted SA and CT for each left hemisphere region were similarly tested using general linear models. This analysis flow was selected such that between-group neuroanatomic analyses and spatial convergence analyses were conducted on the same covariate-adjusted neuroanatomic values. Group differences in regional neuroanatomic measures are shown corrected for multiple comparisons using false discovery rate (FDR) correction (*q*-value < 0.05 across 34 regions).

### Prioritizing 22q11.2 Genes Based on Spatial Convergence of Gene Expression and Neuroanatomic Deviance

To prioritize 22q11.2 genes that may be causally involved in SA or CT alterations in 22q11DS, our primary analysis focused on correlations between regional expression of each brain-expressed, protein-coding 22q11.2 gene and regional deviance in SA and CT, respectively. The neuroanatomic deviance of 22q11DS patients compared with controls was first defined using Z-scores for each covariate-adjusted regional measure of SA and CT. Normalized deviance scores were utilized over raw group difference scores in order to account for differences in the area or average thickness of regions as they are defined in the Desikan-Killiany atlas (i.e., to avoid nonmeaningful larger deviance scores in regions that are defined in the reference Desikan-Killiany atlas as covering a greater number of voxels; Grothe et al. 2018). Thus, mean SA and CT for each left hemisphere region were first calculated for each group. Given the global tendency for 22q11DS patients to show smaller SA overall, mean SA per region for 22q11DS patients was subtracted from the mean for controls and divided by the standard deviation for controls for each region to yield a Z-score severity measure of 22q11DS SA deviance (ΔSA) per region. For CT, given the global tendency for 22q11DS patients to show higher CT, mean CT per region for controls was subtracted from mean CT scores for 22q11DS patients and divided by the standard deviation for controls per region to yield a Z-score severity measure of 22q11DS CT deviance (ΔCT) per region.

Pearson’s correlation coefficients were then used to examine spatial convergence in the expression of each brain-expressed, protein-coding AHBA gene and regional variation in 22q11DS ΔSA and ΔCT severity. Pearson’s correlations were used given that ΔSA, ΔCT, and the expression of the majority of brain-expressed, protein-coding AHBA genes (>75%) were normally distributed across regions (Shapiro–Wilk Test *P* > 0.05) and that these are continuous variables. Spearman’s (nonparametric) correlations yielded highly similar results (see Supplementary Information). Error in correlation coefficient estimates and 95% confidence intervals were assessed with bootstrapping (i.e., resampling the 34 cortical regions 1000 times with replacement) using the “boot” package in R. The ratio of the mean correlation per gene to its bootstrap standard deviation (i.e., Z-score correlation) was used to generate percentile ranks for all brain-expressed, protein-coding genes indexed in AHBA (i.e., 10 344 genes) and to derive corresponding *P*-values based on this empirical distribution of all Z-score correlations across the AHBA. 22q11.2 genes with expression patterns that showed positive spatial correlations with 22q11DS ΔSA or ΔCT severity at extreme high-rank values relative to all brain-expressed, protein-coding AHBA genes (*P*_AHBA_ < 0.05) were considered statistically significant (Forsyth et al. 2020; Seidlitz et al. 2020).

This analysis leverages the fact that the proximal consequence of the 22q11.2 deletion is reduced expression of the majority of deleted genes and assumes that greater severity of neuroanatomic deviation in 22q11DS will be evident in regions where potential causally related genes within the locus are typically most highly expressed.

### Prioritizing 22q11.2 Genes Based on Partial Least Squares Regression

As a complementary approach to the above described primary analysis that leveraged each individual gene and individual SA and CT deviance score per region, we also examined whether an alternate approach utilizing a partial least squares regression (PLSR) data reduction technique would prioritize similar 22q11.2 genes. This secondary analysis was implemented to test the robustness of our primary findings to variation in analysis techniques. Thus, PLSR identifies principal components based on both the predictors (i.e., here, the expression of all brain-expressed, protein-coding genes in AHBA) and the outcome (i.e., here, 22q11DS ΔSA or ΔCT severity) to maximally explain covariance between predictors and the outcome (Wehrens and Mevik 2007). In imaging transcriptomic analyses utilizing PLSR, the first principal component (PLS1) represents the linear combination of gene weights with expression patterns that best predict the neuroimaging measure across regions. PLSR has previously been used to identify biological processes that are broadly associated with spatial neuroanatomic deviance patterns in neuropsychiatric and neurodevelopmental populations (Whitaker et al. 2016; Romero-Garcia 2019; Seidlitz et al. 2018; Morgan et al. 2019) and is generally used in scenarios when prioritizing individual genes out of an a priori set of candidate genes is not the primary analysis goal. Nevertheless, gene weights on the first principal component of a given model can also be used to rank genes with expression patterns that best predict neuroanatomic alterations.

To examine the validity of PLS1 for our ΔSA and ΔCT models, the significance of the variance explained by PLS1 for each model was tested by permuting the outcome labels 10 000 times. To establish gene rankings for PLS1 for each model, error in estimating each gene’s PLS1 weight for the ΔSA and ΔCT models was assessed by bootstrapping (i.e., resampling the 34 cortical regions 1000 times with replacement). The ratio of the mean loading weight of each gene to its bootstrap standard deviation (i.e., Z-score loading) was used to generate percentile ranks for all genes for their PLS1 loading relative to the distribution across all brain-expressed, protein-coding genes in AHBA and derive corresponding empirical *P*-values. Genes with extreme high-rank values (*P*_AHBA_ < 0.05) within this empirical distribution were considered to load significantly on PLS1 for each model. This empirical distribution was also used to prioritize 22q11.2 genes within the PLSR approach. Finally, gene ontology (GO) analyses examined whether all genes that loaded significantly on PLS1 for each model were enriched for specific biological pathways, molecular functions, or cellular components using g:Profiler (Reimand et al. 2016), with “moderate” hierarchical filtering (best per parent) and a minimum query/term overlap size of 5 genes. Only pathways with 10 to 2000 genes were included, and a custom background was set to all protein-coding, brain-expressed AHBA genes.

### Characterizing 22q11.2 Gene Prioritization Relative to Top Genes in Random Gene-Lists

Given that the proximal consequence of 22q11.2 deletions is reduced expression of genes within the locus, our analyses focused on prioritizing individual genes within the locus that are most likely to be causally related neuroanatomic alterations in 22q11DS. Nevertheless, to contextualize the strength of the correlations between the top 22q11.2 genes and ΔSA or ΔCT severity in 22q11DS, we also generated 10 000 random lists of 28 brain-expressed, protein-coding AHBA genes. The correlation Z-scores for the top 22q11.2 gene identified in the primary 22q11SDS ΔSA or ΔCT severity analyses, as well as the Z-score loadings for the top 22q11.2 gene identified in the PLSR analyses for ΔSA or ΔCT severity, were compared with the distribution of top correlation Z-scores or top PLSR Z-score loadings across the 10 000 lists of 28 random genes (*P*_GENE-LIST_).

### Characterizing the Gene Targets of *DGCR8* Deficiency–Induced Down-Regulated miRNAs

Given the prominent gene regulatory role of *DGCR8* via microRNA (miRNA) biogenesis, to follow up on spatial convergence results between regional expression of *DGCR8* and 22q11DS cortical ΔSA severity, we characterized the gene targets of miRNAs previously suggested to be down-regulated in the cortex due to *DGCR8* deficiency. Specifically, given that cortical tissue derived directly from 22q11DS patients was not available, we focused on miRNAs with significantly reduced expression in prefrontal cortex (PFC) in a mouse model of 22q11DS, whose down-regulation was accounted for by *DGCR8* deficiency (Stark et al. 2008). Down-regulated miRNA names were converted from miRBase version 9.1 to miRBase version 21.0 nomenclature using miRNA Accession IDs, and the human gene targets of the homologous human miRNAs were identified using miRTarBase v7.0 (Chou et al. 2018). Gene targets of *DGCR8* deficiency–induced down-regulated miRNAs were functionally annotated using GO biological pathways, molecular functions, and cellular components from g:Profiler (Reimand et al. 2016). Gene targets were also tested for enrichment for lists of genes expressed in specific cell types and specific human brain regions during specific developmental periods (i.e., relative to all other regions/developmental periods) using the Specific Expression Analysis tool (http://genetics.wustl.edu/jdlab/csea-tool-2/;  Dougherty et al. 2010). See Supplementary Material for details.

## Results

### 2‌2q11DS versus Control Differences

The 22q11DS and control groups were similar in age, *F*(1,520) =  0.18, *P* = 0.67, and sex, χ^2^ = 0.98, *P* = 0.32 (Table 1).

**Table 1:** Demographic and summary neuroanatomical characteristics of 22q11DS patients and controls included in the primary analyses

	Control				22q11DS			
	n	Mean age (SD)	n Female (proportion)	Mean ICV mm^3^ (SD)	n	Mean age (SD)	n Female (proportion)	Mean ICV mm^3^ (SD)
Site								
Cardiff	13	14.5 (1.63)	6 (0.46)	1 611 369 (169717)	4	13.8 (0.98)	3 (0.75)	1 480 271 (124686)
Maastricht	38	29.3 (9.62)	15 (0.39)	1 516 538 (213418)	22	30.9 (6.39)	9 (0.41)	1 168 910 (204887)
Newcastle	26	16.8 (3.3)	14 (0.54)	1 674 857 (158963)	10	17.7 (2.5)	7 (0.7)	1 557 987 (168824)
Penn	50	17.5 (3.22)	20 (0.4)	1 568 669 (197479)	40	17.2 (3.24)	17 (0.43)	1 487 143 (195030)
SUNY	19	20.5 (1.24)	8 (0.42)	1 586 112 (197158)	20	20.8 (2.25)	8 (0.4)	1 469 248 (247659)
Toronto1	14	42.4 (8.67)	4 (0.29)	1 559 165 (177790)	11	42.8 (7.28)	6 (0.55)	1 477 699 (168739)
UCDavis1	36	10.4 (2.45)	19 (0.53)	1 535 111 (148718)	23	10.6 (2.04)	10 (0.43)	1 459 221 (154963)
UCDavis2	49	10.8 (2.39)	23 (0.47)	1 561 956 (154247)	49	11.6 (2.56)	25 (0.51)	1 476 262 (183531)
UCLA1	29	14.3 (5.7)	16 (0.55)	1 405 071 (139465)	18	14.4 (5.44)	12 (0.67)	1 373 803 (147130)
UCLA2	16	13.3 (3.6)	5 (0.31)	1 476 150 (145493)	35	15.9 (8.09)	18 (0.51)	1 380 253 (165746)
Total Sample	290	17.8 (9.43)	130 (0.45)	1 547 984 (183491)	232	17.5 (9.12)	115 (0.50)	1 436 108 (198219)

In line with the larger 22q11.2 ENIGMA study (Sun et al. 2020), 22q11DS patients had significantly lower total SA compared with control subjects (22q11DS *M* = 34 665 mm^2^, *SD* = 10 172; Control *M* = 44 437 mm^2^, *SD* = 9409; Z-score ΔSA = −1.04), *F*(1,520) = 129.30, *P* = 2.20 × 10^−16^, and higher mean CT (22q11DS *M* = 3.16 mm, *SD* = 0.11; Control *M* = 3.09, *SD* = 0.11; Z-score ΔCT = +0.64), *F*(1,520) = 57.92, *P* = 1.29 × 10^−13^. Similar to the larger 22q11.2 ENIGMA study, the normalized deviance of SA reductions in 22q11DS was nearly 2-fold in effect size magnitude compared with that for the CT increase in 22q11DS.

**Figure 1:**
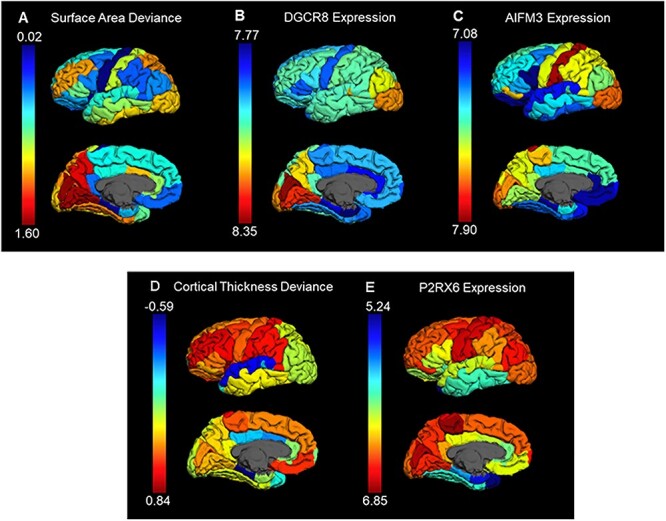
Variation across 34 left hemisphere cortical regions in: A) Z-score surface area deviance (ΔSA) severity in 22q11DS patients relative to controls (higher Z-score indicates region with greater reduction in SA in 22q11DS); B) *DGCR8* expression; C) *AIFM3* expression; D) Z-score cortical thickness deviance (ΔCT) severity in 22q11DS relative to controls (higher Z-score indicates region with greater increase in CT in 22q11DS); and E) *P2RX6* expression. Expression of *DGCR8* and *AIFM3* were significantly associated with ΔSA in 22q11DS and expression of *P2RX6* was significantly associated with ΔCT in 22q11DS.

SA reductions in 22q11DS were widespread, with particularly prominent reductions in midline posterior brain regions, including the cuneus, precuneus, and lingual gyrus, as well as lateral association regions including superior parietal cortex and rostral middle frontal gyrus (Fig. 1A; Supplementary Table 1). Parallel analyses adjusting for ICV identified similar regions of lower SA in 22q11DS versus controls (Supplementary Table 2).

Regional CT differences were also similar to those found in the previous 22q11.2 ENIGMA study, with the majority of regions showing subtle increases in CT in 22q11DS that were greatest in frontal and parietal regions including rostral and caudal middle frontal gyrus, medial and lateral orbitofrontal cortex, precentral and postcentral gyrus, and supramarginal gyrus, as well as in pericalcarine cortex and insula (Fig. 1D; Supplementary Table 3). Significant focal thinning in 22q11DS was found in the caudal anterior cingulate, superior temporal cortex, and parahippocampus.

### Prioritized Genes Associated with Neuroanatomic Alterations in 22q11DS

Spatial correlation analyses comparing the expression patterns of 22q11.2 genes to ΔSA severity in 22q11DS revealed a significant positive correlation between regional expression of *DGCR8* and regional ΔSA severity, Pearson *r* = 0.53, and *P*_AHBA_ = 0.006 (Fig. 1A–C; Table 2). *AIFM3* expression was additionally associated with ΔSA severity, Pearson *r* = 0.42, and *P*_AHBA_ = 0.041. Thus, brain regions with higher expression of *DGCR8* and *AIFM3* in healthy individuals showed greater reductions in cortical SA in 22q11DS (Supplementary Fig. 1). Parallel analyses using SA measures additionally adjusted for ICV, as well as those using Spearman’s correlations, yielded highly similar results (Supplementary Tables 4 and 5, respectively).

**Table 2:** Spatial correlations between expression of 22q11.2 genes and 22q11DS surface area deviance (ΔSA) severity, adjusted for age, sex, and scanner

ΔSA Spatial association rank	Gene	Pearson *r*	Bootstrap Pearson *r* mean (SD)	Bootstrap 95% confidence interval	Bootstrap Pearson *r* Z-score	Pearson *r* Z-score AHBA Rank	Pearson *r* Z-score *P*_AHBA_
**1**	**DGCR8**	**0.53**	**0.52 (0.13)**	**0.21–0.72**	**4.03**	**0.994**	**0.006**
**2**	**AIFM3**	**0.42**	**0.41 (0.14)**	**0.11–0.64**	**2.95**	**0.959**	**0.041**
3	SCARF2	0.30	0.30 (0.17)	−0.07—0.6	1.78	0.833	0.167
4	CLDN5	0.27	0.26 (0.16)	−0.06—0.54	1.62	0.807	0.193
5	DGCR2	0.23	0.23 (0.15)	−0.07—0.51	1.56	0.796	0.204
6	P2RX6	0.22	0.22 (0.15)	−0.10—0.53	1.44	0.778	0.222
7	TANGO2	0.22	0.22 (0.17)	−0.13—0.53	1.31	0.755	0.245
8	RANBP1	0.13	0.13 (0.14)	−0.16—0.37	0.92	0.688	0.312
9	HIRA	0.05	0.06 (0.16)	−0.28—0.36	0.36	0.583	0.417
10	UFD1	0.03	0.02 (0.15)	−0.28—0.29	0.14	0.545	0.455
11	ARVCF	0	0 (0.18)	−0.38—0.35	0.02	0.525	0.475
12	COMT	0	−0.02 (0.19)	−0.44—0.28	-0.12	0.498	0.502
13	MED15	−0.05	−0.06 (0.17)	−0.39—0.31	-0.33	0.461	0.539
14	GNB1L	−0.07	−0.06 (0.15)	−0.34—0.24	-0.42	0.446	0.554
15	PRODH	−0.11	−0.11 (0.18)	−0.45—0.26	-0.62	0.407	0.593
16	SLC25A1	−0.15	−0.16 (0.18)	−0.50—0.20	-0.88	0.355	0.645
17	MRPL40	−0.28	−0.28 (0.17)	−0.59—0.07	-1.70	0.204	0.796
18	GP1BB	−0.25	−0.25 (0.13)	−0.50—0.04	-1.84	0.185	0.815
19	PI4KA	−0.26	−0.26 (0.14)	−0.50—0.02	-1.85	0.184	0.816
20	RIMBP3	−0.33	−0.32 (0.17)	−0.62—0.04	-1.88	0.179	0.821
21	KLHL22	−0.30	−0.29 (0.15)	−0.58—0.02	-1.96	0.166	0.834
22	RTN4R	−0.32	−0.31 (0.15)	−0.59—0.02	-2.09	0.146	0.854
23	C22orf39	−0.40	−0.38 (0.17)	−0.67—0.04	-2.20	0.132	0.868
24	SEPT5	−0.39	−0.39 (0.15)	−0.67—0.05	-2.51	0.095	0.905
25	DGCR6	−0.36	−0.36 (0.12)	−0.59—0.10	-2.89	0.060	0.940
26	DGCR6L	−0.45	−0.45 (0.13)	−0.68—0.16	-3.37	0.031	0.969
27	SNAP29	−0.47	−0.46 (0.12)	−0.67—0.19	-3.76	0.017	0.983
28	SLC7A4	−0.51	−0.51 (0.13)	−0.74—0.21	-3.82	0.016	0.984

Parallel analyses for CT revealed that the spatial pattern of ΔCT severity in 22q11DS was significantly associated with regional expression of *P2RX6*, *r* = 0.43, and *P*_AHBA_ = 0.022 (Fig. 1D-E; Table 3; Supplementary Fig. 2). Thus, regions with higher *P2RX6* expression in healthy individuals showed greater increases in CT among 22q11DS patients compared with healthy controls. Analyses using Spearman’s correlations yielded similar results (Supplementary Table 6).

**Table 3:** Spatial correlations between expression of 22q11.2 genes and 22q11DS CT deviance (ΔCT) severity, adjusted for age, age^2^, sex, and scanner

ΔCT Spatial association rank	Gene	Pearson *r*	Bootstrap Pearson *r* mean (SD)	95% Confidence interval	Bootstrap Pearson *r* Z-score	Pearson *r* Z-score AHBA rank	Pearson *r* Z-score *P*_AHBA_
**1**	**P2RX6**	**0.43**	**0.42 (0.12)**	**0.15–0.64**	**3.46**	**0.978**	**0.022**
2	GNB1L	0.34	0.35 (0.13)	0.09–0.61	2.62	0.931	0.069
3	AIFM3	0.28	0.27 (0.17)	−0.10—0.58	1.54	0.803	0.197
4	TANGO2	0.25	0.25 (0.17)	−0.08—0.56	1.50	0.798	0.202
5	DGCR8	0.24	0.22 (0.16)	−0.11—0.5	1.36	0.774	0.226
6	SCARF2	0.24	0.23 (0.17)	−0.14—0.51	1.37	0.776	0.224
7	RANBP1	0.23	0.23 (0.14)	−0.07—0.49	1.62	0.815	0.185
8	CLDN5	0.19	0.18 (0.14)	−0.10—0.46	1.30	0.764	0.236
9	MRPL40	0.17	0.17 (0.14)	−0.10—0.42	1.22	0.751	0.249
10	DGCR2	0.17	0.18 (0.18)	−0.19—0.50	1	0.713	0.287
11	MED15	0.15	0.14 (0.17)	−0.18—0.46	0.86	0.687	0.313
12	HIRA	0.11	0.10 (0.15)	−0.19—0.38	0.67	0.649	0.351
13	KLHL22	0.09	0.10 (0.19)	−0.27—0.47	0.52	0.622	0.378
14	RTN4R	0.05	0.05 (0.2)	−0.3—0.45	0.25	0.557	0.443
15	COMT	0.04	0.04 (0.15)	−0.30—0.30	0.25	0.558	0.442
16	ARVCF	0.01	0.01 (0.16)	−0.31—0.33	0.05	0.507	0.493
17	UFD1	0.01	0.01 (0.17)	−0.33—0.34	0.07	0.513	0.487
18	RIMBP3	-0.01	0 (0.18)	−0.33—0.38	0.01	0.497	0.503
19	PI4KA	-0.01	−0.01 (0.16)	−0.33—0.31	−0.08	0.475	0.525
20	DGCR6L	-0.10	−0.09 (0.17)	−0.42—0.25	−0.52	0.378	0.622
21	SEPT5	-0.15	−0.14 (0.17)	−0.44—0.19	−0.82	0.317	0.683
22	DGCR6	-0.20	−0.18 (0.15)	−0.45—0.14	−1.22	0.243	0.757
23	GP1BB	-0.22	−0.24 (0.16)	−0.56—0.08	−1.46	0.203	0.797
24	PRODH	-0.27	−0.26 (0.17)	−0.55—0.11	−1.49	0.198	0.802
25	C22orf39	-0.28	−0.26 (0.16)	−0.54—0.09	−1.61	0.177	0.823
26	SLC7A4	-0.31	−0.30 (0.16)	−0.57—0.04	−1.90	0.134	0.866
27	SNAP29	-0.34	−0.33 (0.12)	−0.53—0.08	−2.86	0.041	0.959
28	SLC25A1	-0.36	−0.36 (0.16)	−0.64—0	−2.26	0.088	0.912

Restricting analyses to only brain-expressed, protein-coding genes that were consistently expressed across the 6 AHBA donors (average donor-to-median expression *ρ* > 0.446, as defined by French and Paus (2015); 4947 genes) included 16 22q11.2 genes and similarly identified significant associations between regional expression of *DGCR8* and ΔSA severity in 22q11DS (Supplementary Table 7) and regional expression of *P2RX6* with ΔCT severity (Supplementary Table 8).

### Robustness of Neuroanatomic Deviance in 22q11DS and Prioritized 22q11.2 Genes Across Age Subgroups

To examine the robustness of these relationships across development, we carried out parallel analyses examining group differences and gene expression spatial correlations with ΔSA and ΔCT severity for 3 age subgroups in the 22q11.2 ENIGMA cohort: children (≤12 years; 22q11DS *n* = 75, HC *n* = 89), adolescents (13–17 years; 22q11DS *n* = 67, HC *n* = 77), and adults (≥18 years; 22q11DS *n* = 90, HC *n* = 124). The overall pattern of neuroanatomic differences among 22q11DS patients was highly similar for each age subgroup compared with the overall 22q11DS sample (see Supplementary Tables 9 and 10, respectively). Consistent with this, conducting an exploratory omnibus linear model across age groups, including a CNV group by age group interaction term revealed no significant CNV group by age group interactions for any ROI (FDR corrected *P*-values > 0.05), similar to findings in the larger ENIGMA cohort (Sun et al. 2020). This suggests that neuroanatomic differences in SA and CT in 22q11DS patients are largely established by childhood.

Exploratory spatial correlation analyses for each age group separately revealed significant positive correlations between the expression of *DGCR8* and regional ΔSA severity for all 3 age subgroups (Supplementary Table 11; *r* range: 0.47–0.54, all *P*_AHBAs_ < 0.02). Regional *AIFM3* expression was significantly correlated with ΔSA severity among children (*r* = 0.41, *P*_AHBA_ = 0.029) and adults (*r* = 0.42, *P*_AHBA_ = 0.034), and approached significance in adolescents (*r* = 0.39, *P_AHBA_* = 0.062; Supplementary Table 9). Regional *P2RX6* expression was significantly associated with ΔCT severity among adolescents (*r* = 0.41, *P*_AHBA_ = 0.049) and adults (*r* = 0.53, *P*_AHBA_ = 0.012) and trended toward significance among children (*r* = 0.28, *P*_AHBA_ = 0.078; Supplementary Table 12). *GNB1L* was also associated with ΔCT severity in adolescents (*r* = 0.41, *P*_AHBA_ = 0.044).

### Similar Prioritized 22q11.2 Genes Identified Using Partial Least Squares Regression

Prioritizing 22q11.2 genes based on gene loadings on PLS1 for the 22q11DS ΔSA and ΔCT models highlighted similar genes. Thus, PLS1 for the ΔSA model explained 26.7% of the covariance between regional 22q11DS ΔSA severity and gene expression, which was significantly more than expected by chance (permutation *P* = 0.002). Among the 22q11.2 genes, *DGCR8* and *AIFM3* loaded significantly on PLS1 (*P*_AHBA_ < 0.05; Supplementary Table 13). Functional annotation of all genes with significant loadings on PLS1 indicated that the spatial pattern of ΔSA in 22q11DS was broadly associated with gene regulatory processes, including transcriptional activity and chromatin organization (Supplementary Fig. 3A).

PLS1 for the ΔCT model explained 23.9% of the covariance between regional 22q11DS ΔCT severity and gene expression, which was more than expected by chance (permutation *P* = 0.003). Among 22q11.2 genes, *P2RX6* loaded significantly on PLS1 (*P*_AHBA_ = 0.007; Supplementary Table 14). Functional annotation of all genes with significant loadings on PLS1 indicated that the spatial pattern of ΔCT in 22q11DS was broadly associated with genes involved in transmembrane and ion transmembrane transport (Supplementary Fig. 3B).

### Characterizing 22q11.2 Gene Prioritization Relative to Random Genome-Wide Gene-Lists

Relative to 10 000 lists of 28 random brain-expressed, protein-coding AHBA genes, the strength of the relationship between 22q11DS ΔSA severity and the correlation *Z*-score for the top 22q11.2 gene, *DGCR8*, was greater than the strongest association observed in 85.9% of random gene-lists, *P*_GENE-LIST_ = 0.141. For 22q11DS ΔCT severity, the strength of the relationship with the top 22q11.2 gene, *P2RX6*, was greater than 63.4% of lists, *P*_GENE-LIST_ = 0.366. The top 22q11.2 gene loading on PLS1 for the 22q11DS ΔSA model, *DGCR8*, was similarly greater than for 94.8% of randomly generated gene-lists, *P*_GENE-LIST_ = 0.052, and the top 22q11.2 gene loading for PLS1 for the 22q11DS ΔCT model, *P2RX6*, was greater than for 83.4% of gene-lists, *P*_GENE-LIST_ = 0.166. The elevated but still modest ranking for the strongest 22q11.2 gene spatial correlation with 22q11DS ΔSA and ΔCT severity, respectively, compared with top correlations identified among random sets of any 28 AHBA genes likely reflects the fact that groups of genes work in concert to carry out specific biological functions and, relatedly, that the spatial expression patterns of many genes are highly correlated (Hawrylycz et al. 2012).

### Characterizing Downstream Consequences of *DGCR8* Haploinsufficiency

*DGCR8* was the most prominent gene within the 22q11.2 locus associated with ΔSA severity in 22q11DS. Notably, *DGCR8* is a core component of the miRNA microprocessor complex involved in the biogenesis of miRNAs, which are small noncoding RNAs that critically regulate gene expression by binding target messenger RNA (mRNA) transcripts to accelerate their degradation or silence their translation into proteins. Given this prominent gene regulatory role of *DGCR8*, to better understand mechanisms through which *DGCR8* haploinsufficiency may contribute to ΔSA in 22q11DS, we therefore explored whether gene targets of miRNAs that are down-regulated in mouse PFC due to *DGCR8* deficiency (Stark et al. 2008) converge on specific biological processes.

**Figure 2:**
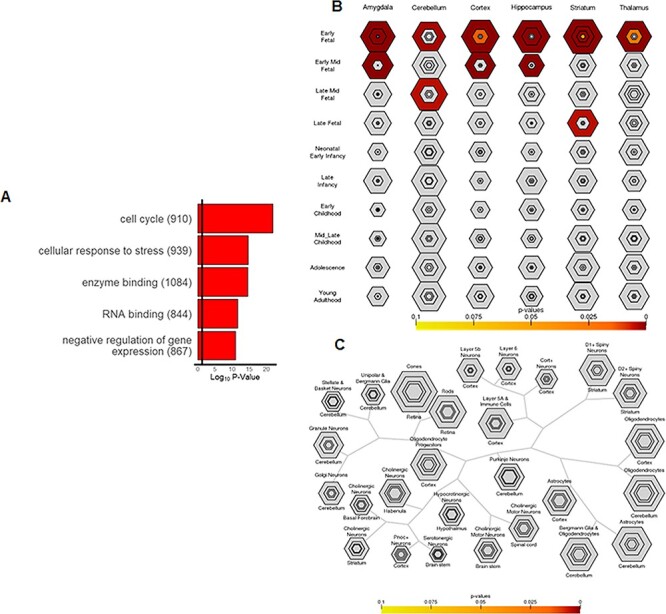
Characterization of 6804 unique gene targets of 59 miRNAs down-regulated in mouse cortex due to *DGCR8* deficiency. A) Top five significantly enriched biological process, molecular function, and cellular component GO terms; B) enrichment for specific developmental periods and brain regions; and C) enrichment for specific CNS cell types, defined at varying specificity indices using the Specific Expression Analysis tool (Dougherty et al. 2010). Varying specificity thresholds in (B) and (C) are represented by the hexagon ring layers going from the least specific gene-lists (outer hexagons) to the most specific gene-lists (center), with hexagons scaled to the size of the gene-lists. BH-corrected Fisher’s Exact *p-*values are plotted for each specificity threshold by color.

The 59 miRNAs down-regulated in PFC (Supplementary Table 15) due to *DGCR8* deficiency targeted 6804 unique human genes (Supplementary Table 16). GO analysis revealed that these genes were enriched for biological processes that include regulation of the cell cycle, cell response to stress, and gene expression (Fig. 2A). *DGCR8*-down-regulated miRNA gene targets were significantly enriched for genes expressed during fetal development across brain regions (Fig. 2B) and were not associated with any specific cell type (Fig. 2C), consistent with this biological pathway enrichment profile. Expanding analyses to to additionally include include gene targets of miRNAs down-regulated in hippocampus due to *DGCR8* deficiency (Stark et al. 2008; Earls et al. 2012) yielded similar results (Fig. S4, Fig. S5).

## Discussion

22q11DS is a rare genetic disorder characterized by neuroanatomic abnormalities that include widespread reductions in cortical SA and thicker cortex overall, with focal thinning in the caudal anterior cingulate, superior temporal cortex, and parahippocampus. As the typically deleted 22q11.2 region (LCR A-D) spans a gene-rich region, identifying which individual genes underlie these neuroanatomic alterations has remained a challenge. Here, by systematically examining the spatial convergence between severity of cortical SA and CT deviation in 22q11DS and expression of each 22q11.2 gene, we provide novel evidence prioritizing *DGCR8* as a potential contributor to pervasive SA reductions in 22q11DS. *AIFM3* was also associated with 22q11DS SA reductions. Interestingly, *P2RX6* was associated with ΔCT, suggesting that this gene may contribute to increases in CT in individuals with 22q11DS. Notably, regional expression of *DGCR8* was robustly associated with severity of ΔSA in 22q11DS across all age subgroups examined, suggesting that *DGCR8* haploinsufficiency may disrupt early aspects of corticogenesis that are established by childhood.

*DGCR8* is essential for the biogenesis of miRNAs, which regulate gene expression at the protein level (Rajman and Schratt 2017), and has previously been suggested to play a key role in 22q11DS phenotypes (Stark et al. 2008; Earls et al. 2012; Merico et al. 2014; Eom et al. 2020; Forsyth et al. 2020). Specifically, *DGCR8* forms a complex with Drosha to cleave long primary miRNAs (pri-miRNAs) into short precursor miRNAs (~70 nucleotides in length), before they are cleaved into mature miRNAs (~22 nucleotides in length) by Dicer. Mature miRNAs are then loaded into the RNA-induced silencer complex (RISC), where they serve as a guide RNA strand for binding target mRNA transcripts to silence their translation into proteins. miRNA activity is increasingly recognized as a powerful posttranscriptional gene regulatory mechanism for diverse cellular processes, and in the brain, it is known to play a particularly important role in regulating cell proliferation, growth, and differentiation during fetal development (Yao et al. 2012; Rajman and Schratt 2017). Our finding that the gene targets of miRNAs down-regulated in mouse cortex due to *DGCR8* deficiency are enriched for fetal-specific expression and regulation of the cell cycle and cell proliferation is consistent with these known roles of miRNAs. Notably, cell cycle parameters govern the balance between stem cell proliferation and differentiation during early brain development and thereby critically modulate cortical size and structure (Rakic 2009). In line with this, while knockout of *DGCR8* is lethal, conditional knockout of *DGCR8* in mouse embryonic stem (ES) cells has been found to disrupt cell cycle progression, ES cell proliferation, and differentiation (Wang et al. 2007). Similarly, knockout of *DGCR8* in neural progenitors was found to disrupt progenitor pool maintenance, differentiation, and corticogenesis (Marinaro et al. 2017). Knockout of *DGCR8* in pyramidal neurons also resulted in severe microcephaly, cell loss, altered inhibitory synaptic transmission, and premature death (Hsu et al. 2012). Although hemizygous depletion of *DGCR8* yields milder effects on miRNA biogenesis relative to *DGCR8* knockout, with some differences in brain phenotypes (Marinaro et al. 2017), *DGCR8^+/−^* mice have been found to have altered cell proliferation, neurogenesis, and neuronal morphology (Stark et al. 2008; Fénelon et al. 2011; Ouchi et al. 2013; Amin et al. 2017), as well as enlarged brain ventricular volumes (Eom et al. 2020), altered homeostatic and synaptic plasticity (Earls et al. 2012; Amin et al. 2017), and cognitive deficits (Stark et al. 2008; Fénelon et al. 2011; Ouchi et al. 2013). Human induced pluripotent stem cell–derived neurons with hemizygous *DGCR8* loss also show changes in calcium signaling and excitability (Khan et al. 2020). Our finding that *DGCR8* expression is robustly associated with severity of SA deviation in 22q11DS across age subgroups is consistent with this literature implicating *DGCR8* as a key contributor to brain and behavioral phenotypes in 22q11DS, and suggests that *DGCR8* hemizygosity may contribute to SA reductions by disrupting miRNA modulation of cell cycle regulation during early brain development in 22q11DS.

*AIFM3* was also associated with SA deviation in 22q11DS. *AIFM3* is a proapoptotic protein that appears to stimulate cell death by depolarizing the membrane potential of mitochondria and activating the classical caspase-dependent apoptotic cascade (Xie et al. 2005; Zheng et al. 2019). Notably, in addition to the fundamental role of cell cycle regulation in defining the size and structure of the cortex, apoptosis also critically regulates cortex size (Haydar et al. 1999). Thus, two prominent waves of apoptosis are known to occur during corticogenesis. The first wave involves the elimination of a large number of dividing neuronal precursor cells during the peak of neurogenesis, regulating the size of the neuronal precursor pool. The second wave involves the elimination of postmitotic neurons during neuronal migration, regulating the wiring of developing neuronal networks (Blomgren et al. 2007). Although little is known about the specific role of *AIFM3* in brain development, *AIFM3* is highly expressed in the human brain (https://gtexportal.org/home/), supporting the possibility that *AIFM3* haploinsufficiency could contribute to SA deficits by altering normal apoptotic processes during corticogenesis.

Finally, *P2RX6* was associated with CT deviation in 22q11DS. *P2RX6* encodes the P2X6 receptor—a member of the P2X-purinergic family of ATP-gated ion channels that mediates fast excitatory postsynaptic potentials in neurons and smooth muscles (Motahari et al. 2019) and exerts neuromodulatory functions (Khakh and North 2012). While little is known about the role of *P2RX6* during brain development, it is expressed in both developing and adult brain and is alternately spliced in the developing mouse brain and during in vitro neuronal differentiation (da Silva et al. 2007). Although speculative, *P2RX6* deficiency could contribute to CT abnormalities in 22q11DS by altering modulation of synaptic signaling. Additional work is needed to experimentally validate that *P2RX6* deficiency contributes to abnormalities in CT in 22q11DS and clarify underlying mechanisms.

Some potential limitations to the present study should be noted. First, our analyses are correlational in nature and assume causal, independent effects of deficient expression of one or more 22q11.2 genes on the regional severity patterns of SA and CT alterations in 22q11DS. Although the 22q11.2 deletion is known to yield significantly reduced expression of the majority of genes in the region (Stark et al. 2008; Jalbrzikowski et al. 2015; Lin et al. 2016; Gordon et al. 2019), experimental validation is necessary to confirm the present prioritization of *DGCR8*, *AIFM3,* and *P2RX6* as genes that may be causally related to cortical alterations in 22q11DS. In addition, our analyses do not address the possibility that interactions between genes within the locus or with additional downstream interacting partners may also contribute to neuroanatomic alterations in 22q11DS, nor for the possibility that some brain abnormalities in 22q11DS may arise in part as downstream consequences of abnormalities in other organs, such as heart anomalies, which could be mediated by hemizygosity of genes within the 22q11.2 locus that are not expressed in brain (Schaer et al. 2009, 2010). In line with this, the strongest correlations (e.g., for spatial convergence between *DGCR8* expression and ΔSA) were moderate in strength, indicating that some variability in regional SA and CT deviance is not predicted by individual expression of these genes, as captured by the AHBA. Whether this is due to measurement error, interactions between genes and/or with downstream interacting partners, nonuniform effects of hemizygosity of individual 22q11.2 genes on corticogenesis in different brain regions, or other explanations remains to be determined. Additionally, we lacked high spatial resolution gene expression data from the developing human brain when SA and CT are initially shaped. We therefore relied on spatial expression data from the AHBA to derive insight into molecular mechanisms underlying neuroimaging phenotypes in 22q11DS, similar to other groups that have leveraged the AHBA to understand neuroanatomic abnormalities in neuropsychiatric, neurodevelopmental, and neurodegenerative disorders (Rittman et al. 2016; Romme et al. 2017; Romero-Garcia 2019; Fornito et al. 2019). Given these limitations, negative findings should be interpreted with caution, and future studies using gene expression data from developing brain samples collected at high spatial resolution will be important to confirm the present findings. Finally, our exploratory analysis of down-regulated miRNA gene targets due to *DGCR8* hemizygosity was based on findings in postnatal cortical samples from 22q11.2- and *DGCR8*-deficient mouse models; it would be ideal to examine down-regulated miRNAs in cortex derived directly from individuals with 22q11DS or with *DGCR8*-specific hemizygous mutations, particularly during early corticogenesis. In the absence of such tissue, we nevertheless believe it is useful to identify biological pathways associated with gene targets of miRNAs previously identified as down-regulated in cortex due to *DGCR8* deficiency. Conversely, the large sample of individuals with confirmed A-D deletions and matched control subjects used to derive spatial measures of SA and CT deviation is a relative strength of the present study.

In summary, by integrating comprehensive maps of genome-wide gene expression in the human brain and neuroanatomic data from the largest existing sample of 22q11DS individuals with molecularly confirmed A-D deletions, we prioritized *DGCR8* and *AIFM3* as potential contributors to cortical SA alterations in 22q11DS, and *P2RX6* as a potential contributor to CT alterations. While *DGCR8* deficiency has been found to modulate cell cycle progression, neural progenitor differentiation, and corticogenesis in animal models (Wang et al. 2007; Marinaro et al. 2017; Hoffmann et al. 2018), experimental validation is needed to confirm that *AIFM3* and *P2RX6* deficiency yield abnormalities in cortical SA and thickness, respectively. Nevertheless, *DGCR8* and *AIFM3* are involved in regulating two neurodevelopmental processes thought to fundamentally modulate brain size and structure (i.e., cell proliferation and apoptosis, respectively). Our finding that systematic investigation of 22q11.2 genes prioritized relatively understudied genes (i.e., *AIFM3* and *P2RX6*) and a gene previously hypothesized to play a key role in 22q11.2 phenotypes (i.e., *DGCR8*), provides important opportunities to pursue novel mechanistic hypotheses in 22q11DS. Together, these results demonstrate the utility of combining neuroanatomic and publicly available transcriptomic datasets to derive mechanistic insights and prioritize individual genes that may underlie neuroanatomic differences in multigene CNVs.

## Funding

This work was supported by the following funding sources: Anillo PIA ACT192064 from Comisión Nacional de Investigación Científica y Tecnológica (CONICYT) Chile (Crossley); Canadian Institutes of Health Research (CIHR) MOP-79518 (Bassett); CIHR MOP-111238 (Bassett); CIHR MOP-74631 (Chow); CIHR MOP-89066 (Bassett); CIHR MOP-97800 (Bassett); Dalglish Family Chair in 22q11.2 Deletion Syndrome (Bassett); Dutch Brain Foundation 15F07(2).55 (Bakker, Amelsvoort); Dutch Organisation for Health Research and Development ZonMW-Veni grant 2006 916.76.048 (Bakker, Amelsvoort); European Autism Interventions (EU-AIMS)/EU AIMS-2-TRIALS, a European Innovative Medicines Initiative Joint Undertaking grants 115300 and 777394 (D.G. Murphy); FONDECYT Chile 1160736 (Crossley); FONDECYT Chile 1171014 (Repetto); HDU54079125 (Simon); King’s College London (D.G. Murphy, C.M. Murphy); Lifespan Brain Institute (Gur, Roalf); Maudsley NHS Foundation Trust (D.G. Murphy); Michael Smith Foundation for Health Research and the Seedlings Foundation (Vila-Rodriguez); MRC Centre MR/L010305/1 (Cunningham, Moss, Owen, van den Bree); NARSAD Young Investigators Awards (Forsyth, Roalf); National Center for Advancing Translational Sciences UCLA CTSI Grant UL1TR001881 (Bearden, Forsyth); National Institute for Health Research Maudsley Biomedical Research Centre at South London Maudsley Foundation Trust (C.M. Murphy); National Institute for Health Research Mental Health Biomedical Research Centre at South London (D.G. Murphy); National Institute of Child Health and Human Development (NICHD) PO1-HD070454 (Crowley, Emanuel, McDonald-McGinn, Zackai); National Institute on Aging (NIA) T32AG058507 (Ching); NIA R56AG058854 (Thompson); NIH/National Institute of Biomedical Imaging and Bioengineering (NIBIB) U54EB020403 from the Big Data to Knowledge (BD2K) Program (Bearden, Ching, Thompson); NIBIB P41EB015922 (Thompson); NIH R01HD042794 (Simon); NIH R01MH107108 (Simon); NIH UO1-MH191719 (Crowley, Emanuel, McDonald-McGinn, Zackai); National Institute of General Medical Sciences R01 GM125757 (Emanuel); National Institutes of Mental Health (NIMH) K08MH118577 (Forsyth); NIMH 5T32MH073526 (Ching); NIMH MH064824 (Kates); NIMH R01MH085953 (Bearden); NIMH R01MH087636-01A1 (Crowley, Emanuel, McDonald-McGinn, Zackai); NIMH R01MH100900 (Bearden); NIMH R01MH111671 (Thompson); NIMH R01MH116147 (Thompson); NIMH R01MH119185 (Roalf); NIMH U01MH087626 (Gur); NIMH U01MH101719 (Bearden, Gur); NIMH U01MH101719-04S1 (Bearden); NIMH U01MH101723-01(3/5) (Bassett, Chow); NIMH U01MH101724 (van den Bree); NIMH U01MH119738 (Gur, van den Bree); Wellcome Trust 100202/Z/12/Z (Doherty, Linden, Owen); Wellcome Trust Institutional Strategic Support Fund (ISSF) Award (van den Bree).

### Notes

Drs Ching and Thompson have received partial research support from Biogen, Inc. (Boston, USA) for work unrelated to the topic of this manuscript. Dr Antshel has received investigator-initiated research funds from Shire and has participated in an advisory panel for Arbor Pharmaceuticals. Dr D.G. Murphy has served on an advisory board for Roche. Dr Vila-Rodriguez has received research support from Brain Canada, the Canadian Institutes of Health Research, the Michael Smith Foundation for Health Research, and the Vancouver Coastal Health Research Institute and in-kind equipment support from MagVenture, and he has served on an advisory board for Janssen. Dr Linden receives editorial fees from Elsevier and book royalties from Springer Nature and Oxford University Press. Dr Owen has received research support from Takeda. Dr van den Bree has received research support from Takeda. Dr Thompson has received grant support from Biogen. The other authors report no financial relationships with commercial interests.

### 2‌2q11.2 ENIGMA Consortium Authors

Rachel K. Jonas, PhD, Laura Pacheco-Hansen, MA, Geor Bakker, PhD, Therese van Amelsvoort, MD, PhD, Kevin M. Antshel, PhD, Wanda Fremont, MD, Wendy R. Kates, PhD, Linda E. Campbell, PhD, Kathryn L. McCabe, PhD, Michael C. Craig, MD, Eileen Daly, PhD, Maria Gudbrandsen, MSc, Clodagh M. Murphy, MD, PhD, Declan G. Murphy, MD, FRC Psych., Kieran C. Murphy, MD, PhD, Ania Fiksinski, MSc, Sanne Koops, PhD, Jacob Vorstman, MD, PhD, T. Blaine Crowley, BA, Beverly S. Emanuel, PhD, Raquel E. Gur, MD, PhD, Donna M. McDonald-McGinn, MS, LCGC, David R. Roalf, PhD, Kosha Ruparel, MSE., J. Eric Schmitt, MD, PhD, Elaine H. Zackai, MD, Courtney A. Durdle, BA, BS, Naomi J. Goodrich-Hunsaker, PhD, Tony J. Simon, PhD, Anne S. Bassett, MD, Nancy J. Butcher, PhD, Eva W.C. Chow, MD, MPH, Fidel Vila-Rodriguez, MD, PhD, Adam Cunningham, PhD, Joanne L. Doherty, PhD, David E. Linden, PhD, Hayley Moss, MSc, Michael J. Owen, MD, PhD, Marianne van den Bree, PhD, Nicolas A. Crossley, MD, PhD, and Gabriela M. Repetto, MD.

### 2‌2q11.2 ENIGMA Consortium Author Affiliations

Department of Psychiatry and Biobehavioral Sciences, Semel Institute for Neuroscience and Human Behavior, Los Angeles, CA, USA (Forsyth, Mennigen, Lin, Sun, Jonas, Pacheco-Hansen, Vajdi, Bearden); Department of Psychology, UCLA, Los Angeles, CA, USA (Bearden); Interdepartmental Neuroscience Program, UCLA, Los Angeles, CA,USA (Lin, Jonas); Imaging Genetics Center, Mark and Mary Stevens Institute for Neuroimaging and Informatics, Keck School of Medicine, Los Angeles, CA, USA (Ching, Villalon Reina, Thompson); Department of Psychiatry and Neuropsychology, Maastricht University, Maastricht, the Netherlands (Bakker, van Amelsvoort); Department of Radiology and Nuclear Medicine, Amsterdam University Medical Centers, Amsterdam, the Netherlands (Bakker); Department of Psychology, Syracuse University, Syracuse,NY,USA (Antshel); Department of Psychiatry and Behavioral Sciences, SUNY Upstate Medical University, Syracuse, NY, USA (Fremont, Kates); School of Psychology, University of Newcastle, Newcastle, Australia (Campbell,McCabe); MIND Institute and Department of Psychiatry and Behavioral Sciences, University of California Davis, Davis, CA, USA (McCabe, Durdle, Goodrich-Hunsaker, Simon); Institute of Psychiatry, Psychology, and Neuroscience, Sackler Institute for Translational Neurodevelopment, and Department of Forensic and Neurodevelopmental Sciences, King’s College London, London, UK (Craig, Daly, Gudbrandsen, C.M. Murphy, D.G. Murphy); Bethlem Royal Hospital, National Institute for Health Research Maudsley Biomedical Research Centre, and SLaM NHS Foundation Trust, National Autism Unit, London, UK (Craig); Behavioral Genetics Clinic, Adult Autism Service, Behavioral and Developmental Psychiatry Clinical Academic Group, South London and Maudsley NHS Foundation Trust, London, UK (C.M. Murphy, D.G. Murphy); Department of Psychiatry, Royal College of Surgeons in Ireland, and Education and Research Centre, Beaumont Hospital, Dublin, Ireland (K.C. Murphy); Department of Psychiatry, Brain Center Rudolf Magnus, University Medical Center Utrecht, Utrecht, the Netherlands (Fiksinski, Koops, Vorstman); Clinical Genetics Research Program (Bassett, Chow, Fiksinski) and Clinical Genetics Service (Chow), Centre for Addiction and Mental Health, Toronto, ON, Canada; Dalglish 22q Clinic for Adults, Toronto General Hospital, Toronto, ON, Canada (Bassett, Fiksinski); Department of Psychiatry, University of Toronto, Toronto, ON, Canada (Bassett, Butcher, Chow, Vorstman); Department of Neurology, University of Utah, Salt Lake City, Utah (Goodrich-Hunsaker); Program in Genetics and Genome Biology, Research Institute, and Department of Psychiatry, Hospital for Sick Children, Toronto, ON, Canada (Vorstman); Division of Human Genetics and 22q and You Center, Children’s Hospital of Philadelphia, Philadelphia, PA, USA (Crowley, Emanuel, McDonald-McGinn, Zackai); Department of Pediatrics, University of Pennsylvania Perelman School of Medicine, Philadelphia, PA, USA (Emanuel, McDonald-McGinn, Zackai); Department of Psychiatry, University of Pennsylvania Perelman School of Medicine and Children’s Hospital of Philadelphia, PA, USA (Gur); Department of Psychiatry, University of Pennsylvania Perelman School of Medicine, Philadelphia, PA, USA (Roalf, Ruparel); Departments of Radiology and Psychiatry, Hospital of the University of Pennsylvania, Philadelphia, PA, USA (Schmitt); Department of Psychological and Brain Sciences, University of California, Santa Barbara, CA, USA (Durdle); Child Health Evaluative Sciences, Hospital for Sick Children Research Institute, Toronto, ON, USA (Butcher); Department Psychiatry, University of British Columbia, Vancouver, BC, Canada (Vila-Rodriguez); MRC Centre for Neuropsychiatric Genetics and Genomics, Division of Psychological Medicine and Clinical Neurosciences, Cardiff, University, Cardiff, UK (Cunningham, Doherty, Linden, Moss, Owen, van den Bree); Cardiff University Brain Research Imaging Centre, Cardiff University, Cardiff, UK (Doherty, Linden); Department of Psychiatry, Pontificia Universidad Católica de Chile, Santiago, Chile (Crossley); Clinica Alemana, Universidad del Desarrollo, Centro de Genética y Genomica, Facultad de Medicina, Santiago, Chile (Repetto); Departments of Neurology, Psychiatry, Radiology, Engineering, Pediatrics, and Ophthalmology, University of Southern California, Los Angeles, CA, USA (Thompson).

## Supplementary Material

CerCor-2020-00720_SuppInfo_121420_bhab008

SupplementaryTables_15_16_17_miRNA_Analysis_111220_bhab008
