# Supplementary material for: Prioritizing Genetic Contributors to Cortical Alterations in 22q11.2 Deletion Syndrome Using Imaging Transcriptomics
Source: Cereb Cortex. 2021 Feb 26;31(7):3285–98. doi: 10.1093/cercor/bhab008 (PMC8196250; doi:10.1093/cercor/bhab008)
Supplement: CerCor-2020-00720_SuppInfo_121420_bhab008 [file cercor-2020-00720_suppinfo_121420_bhab008.docx]

**Supplementary Material**

**Robustness of Neuroanatomic 22q11DS Deviance Across Age Subgroups**

The overall pattern of neuroanatomic differences among 22q11DS patients was highly similar for each age subgroup compared to the overall 22q11DS group. Thus, across age groups, 22q11DS patients showed widespread SA reductions that were particularly prominent in parietal-occipital regions (Table S9), as well as increases in many regions in CT that were prominent in frontal and parietal regions (Table S10). Focal thinning in 22q11DS was significant in each age subgroup in the parahippocampal gyrus; was significant in the superior temporal gyrus among adolescents and adults but did not survive FDR correction in children; and was not significant in the caudal anterior cingulate for any age group, although the Z-score deviance magnitude was similar in each age subgroup compared to the overall sample. Together, this suggests that neuroanatomic differences in SA and CT in 22q11DS patients are largely established by childhood.

**Characterizing Downstream Consequences of DGCR8 Haploinsufficiency**

To follow up on spatial convergence results between regional expression of *DGCR8* and 22q11DS ΔSA severity, our primary analysis characterized the gene targets of miRNAs down-regulated in the cortex of a mouse model of 22q11DS, whose down-regulation was accounted for by *DGCR8* deficiency (see Stark et al., 2008 for details). Briefly, Stark et al., (2008) assessed the expression levels of 15 pri-miRNAs and 386 mature miRNAs in the prefrontal cortex (PFC) of 8-week old mice engineered to have a 1.3 Mb heterozygous deletion syntenic to a ~1.5 Mb A-B deletion at the 22q11.2 locus that includes *DGCR8* (Df(16)A^+/-^ mice). Pri-form-specific qRT-PCR was used to assay the expression of pri-miRNAs and mature miRNA expression levels were assayed using the mirVana miRNA isolation kit using probe sequences based on miRBase sequence database version 9.1. The expression of a subset of pri-miRNAs and mature miRNAs was then assessed in mice engineered to have reduced expression of *DGCR8*, specifically (*DGCR8*^+/-^ mice). As identical patterns of dysregulation in PFC were found among the pri-miRNAs and mature miRNAs assayed in both the *DGCR8*^+/-^ and Df(16)A^+/-^ mice, miRNA dysregulation found in Df(16)A^+/-^ mice was concluded to be due to *DGCR8* haploinsufficiency.

Fifty nine mature miRNAs had significantly reduced expression in the PFC of Df(16)A^+/-^ mice and are listed in Table S15; miRNA names were converted from miRBase version 9.1 to miRBase version 21.0 nomenclature using miRNA Accession IDs. The human gene targets of the homologous human miRNAs were identified using miRTarBase v7.0 (Chou et al., 2018). miRTarBase is a centralized repository of miRNA-target interactions (MTI) that have been experimentally validated by various approaches such as reporter assays, western blot, or microarray experiments. miRTarBase v7.0 contains 422,517 MTI between 4076 miRNAs and 23,054 genes across species, collected from 8,573 articles. MTIs supported by strong (i.e. reporter assay and/or western blot) versus more limited experimental evidence (e.g. HITS-CLIP) are also annotated.

Down-regulated miRNA gene targets were functionally annotated using gene ontology (GO) biological pathways, molecular functions, and cellular components from g:Profiler [(rev 1760, Reimand *et al.*, 2007)](https://paperpile.com/c/YtRzqs/oog6O) with “moderate” hierarchical filtering (best per parent) and a minimum query/term overlap size of five genes. Only pathways with 10 to 2000 genes were included in the analyses. A custom background was set to all unique human gene targets in miRTarBase (i.e. 15,064 genes). Top enriched terms surpassing g:SCS (Set Counts and Sizes) p < 0.05 genes are shown. Down-regulated miRNA gene targets were also tested for enrichment for lists of genes expressed in specific human brain regions during specific developmental periods (i.e., relative to all other regions/developmental periods) using the Specific Expression Analysis tool (<http://genetics.wustl.edu/jdlab/csea-tool-2/>; Dougherty et al., 2010). The Specific Expression Analysis tool implements BH-corrected Fisher’s Exact tests to test enrichment for their specific expression gene-lists, which were defined using Specificity Index thresholds (pSI) of varying stringency (e.g., pSI < 0.01 involves a larger number of transcripts with relative enrichment for specific brain regions during specific developmental periods, whereas pSI < 0.0001 involves a smaller subset of transcripts with highly specific expression). The web-based tool provides graphical “bullseye” plots summarizing the enrichment of input gene-lists for the region and developmental period specific gene-sets, across the varying pSI thresholds. As described in Dougherty et al., 2010, the region and developmental period specific gene-lists were derived from RNA-seq BrainSpan data aggregated into 6 major regional divisions across 10 developmental periods.

We additionally extended these analyses to incorporate miRNAs that were significantly down-regulated in the hippocampus of 8-week old Df(16)A^+/-^ mice in Stark et al., (2008), and accounted for by DGCR8 haploinsufficiency, or in the hippocampus of 16-week old Df(16)1/+ mice in Earls et al., (2012). Earls et al., (2012) used mouse miRNA microarray to assess the expression of 690 miRNAs based on miRBase v14.0 sequences in mice engineered to have a hemizygous deletion of 23 genes in a region of chromosome 16 that is syntenic to the human 22q11.2 locus and includes *DGCR8* (Df(16)1/+ mice). qPCR was used to verify expression changes in a subset of miRNAs in Df(16)1/+ mice and DGCR8+/- mice. As in Stark et al., (2008), among those miRNAs that were assessed by qPCR, DGCR8+/- mice showed a similar pattern of miRNA expression changes as Df(16)1/+ mice (Earls et al. 2012), suggesting that DGCR8 haploinsufficiency likely also underlies the broader miRNA expression changes observed via microarray in the hippocampus of Df(16)1/+ mice. miRNA names from Earls et al., (2012) were similarly converted from miRBase version 14.0 to miRBase version 21.0 nomenclature using miRNA Accession IDs. Human gene targets of the homologous human miRNAs were identified using miRTarBase v7.0 (Chou et al., 2018). Pooling down-regulated miRNAs from hippocampal tissue from these two sources with downregulated miRNAs from the PFC identified 94 unique miRNAs in miRBase v21 that were significantly downregulated in these mouse models in cortex or hippocampus (Table S15), with 8,428 experimentally validated, unique gene targets identified in miRTarBase v7.0 (Table S16). Functional characterization of this larger pool of gene targets yielded highly similar results (Figure S4A-C) compared to analyses restricted to miRNAs downregulated due to DGCR8 haploinsufficiency in mouse cortex, specifically. Similarly, while initial analyses characterized gene targets based on any experimental evidence; functional characterization of gene targets derived only from strong experimental evidence (904 genes; Table S17) yielded highly similar results (Figure S5A-C).

**Figure S1.** Scatterplots of spatial correlation between Z-score surface area deviance (ΔSA) severity in 22q11DS and expression of (A) *DGCR8* and (B) *AIFM3*.

**A**


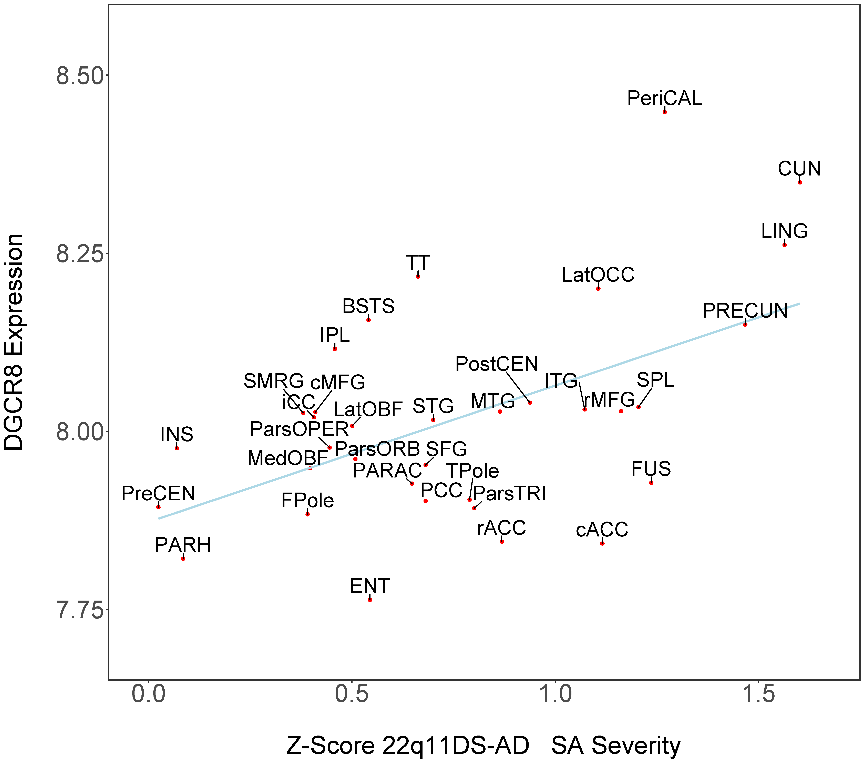


Δ

*r* = 0.53, *p* = 0.006

Z-Score 22q11DS ΔSA Severity

**B**


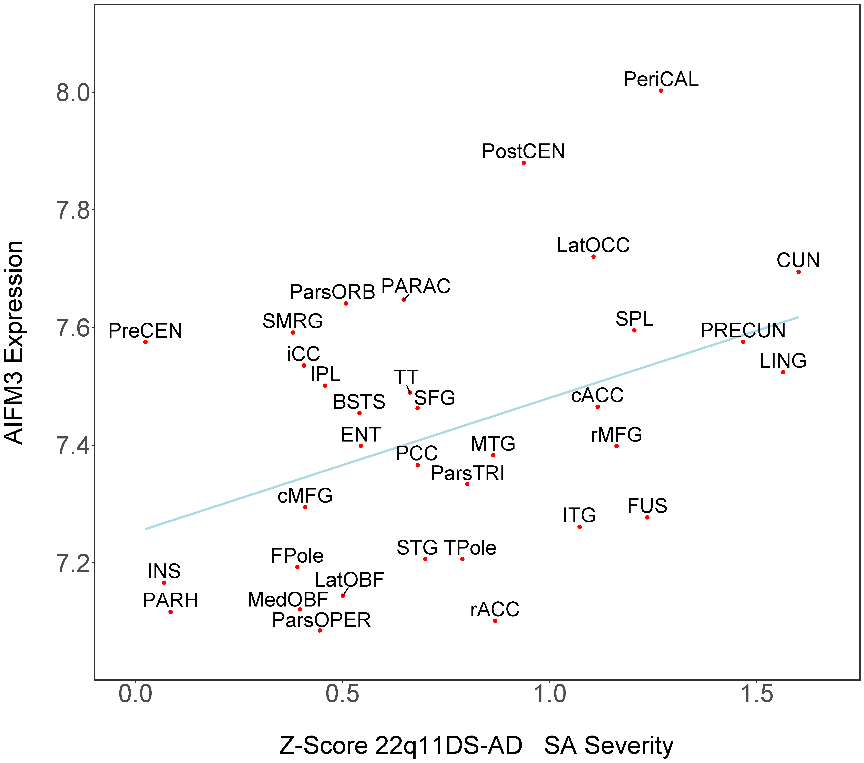


Δ

*r* = 0.42, *p* = 0.041

Z-Score 22q11DS ΔSA Severity

**Figure S2.** Scatterplots of spatial correlation between Z-score cortical thickness deviance (ΔCT) severity in 22q11DS and expression of *P2RX6*.


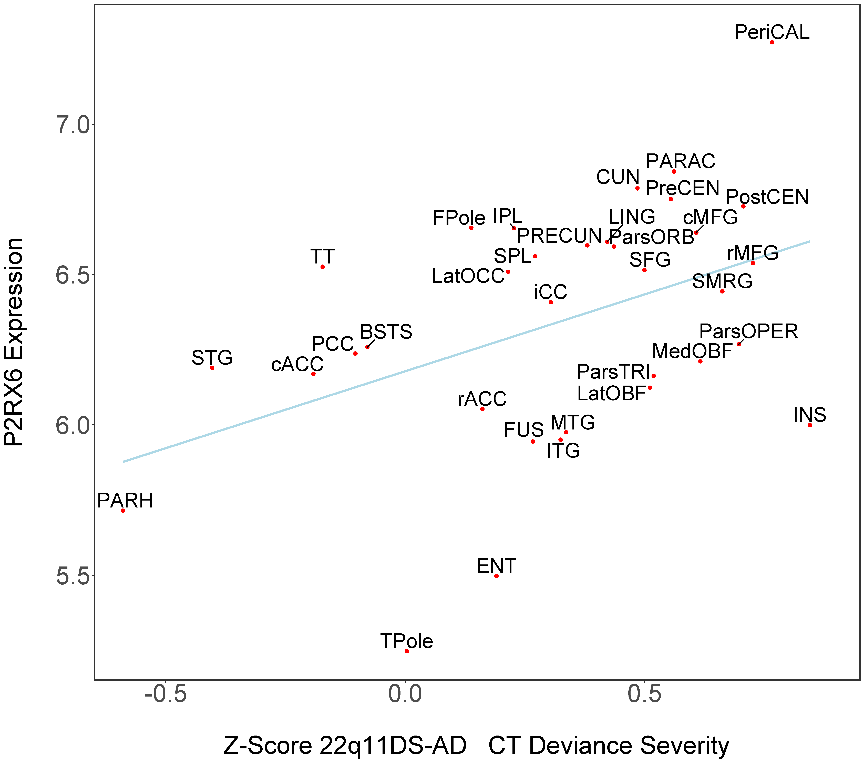


Δ

*r* = 0.43, *p* = 0.022

Z-Score 22q11DS ΔCT Severity

**Figure S3.** Top significantly enriched biological process, molecular function, and cellular component gene ontology (GO) terms (up to 5) for genes that loaded significantly on: A) PLS1 for 22q11DS surface area deviance (ΔSA) severity model, and B) PLS1 for 22q11DS cortical thickness deviance (ΔCT) severity model.


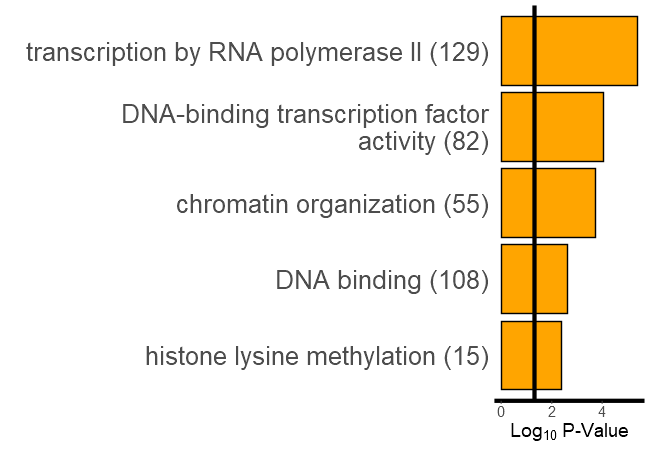


B

A


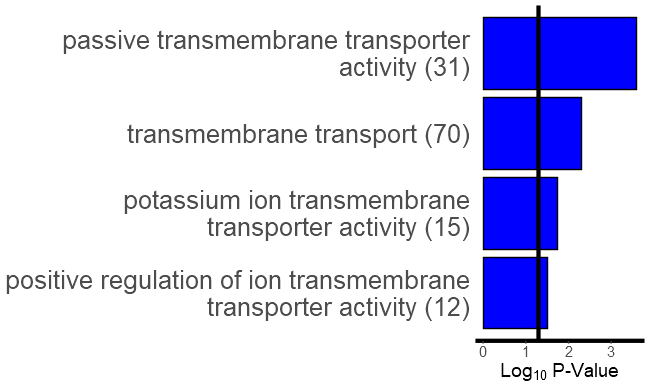


**Figure S4.** Characterization of 8,428 unique gene targets of 94 miRNAs found to be downregulated in mouse prefrontal cortex or hippocampus due to *DGCR8* deficiency. A) Top five significantly enriched biological process, molecular function, and cellular component gene ontology (GO) terms; B) enrichment for specific developmental periods and brain regions; and C) enrichment for specific CNS cell types, defined at varying specificity indices using the Specific Expression Analysis tool [(Dougherty *et al.*, 2010)](https://paperpile.com/c/YtRzqs/vk47t). Varying specificity thresholds in (B) and (C) are represented by the hexagon ring layers going from the least specific gene lists (outer hexagons) to the most specific gene lists (center), with hexagons scaled to the size of gene lists. BH corrected Fisher’s Exact *p* values are plotted for each specificity threshold by color.


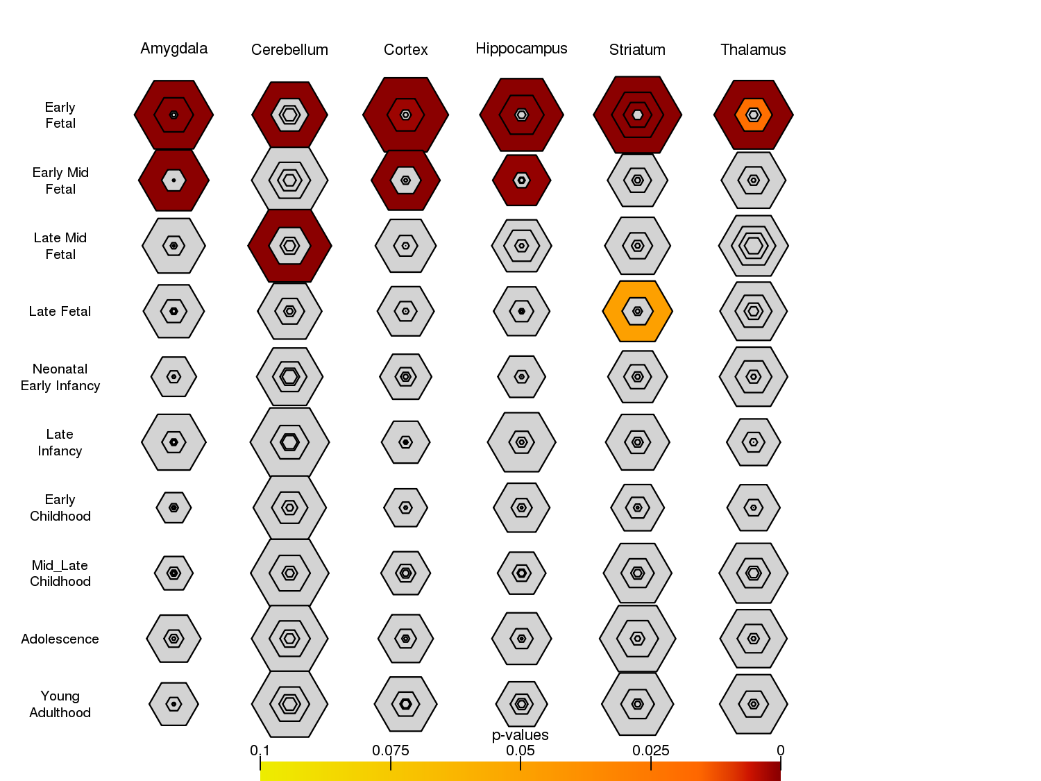


**B**


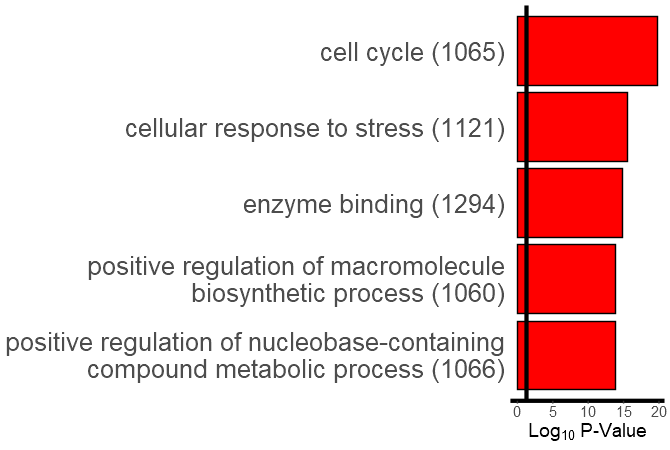


**A**


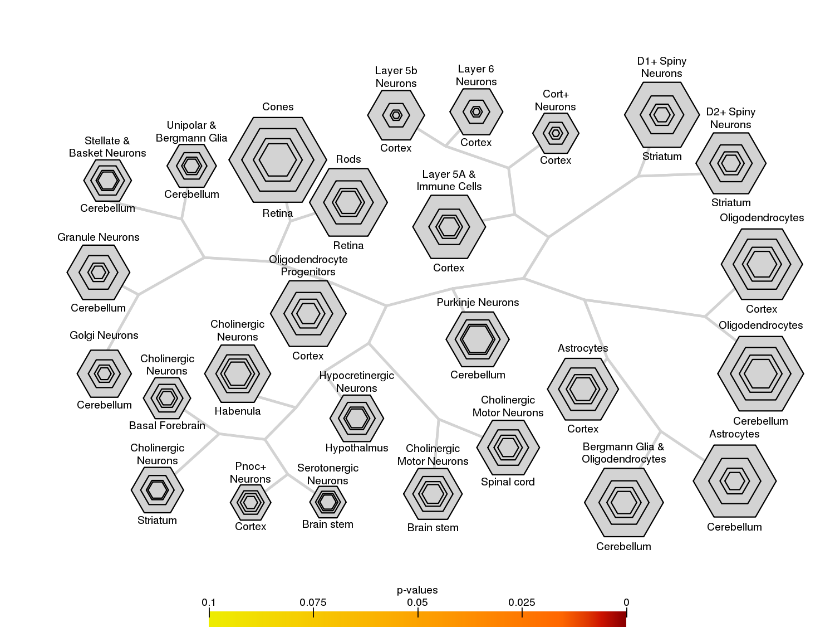


**C**


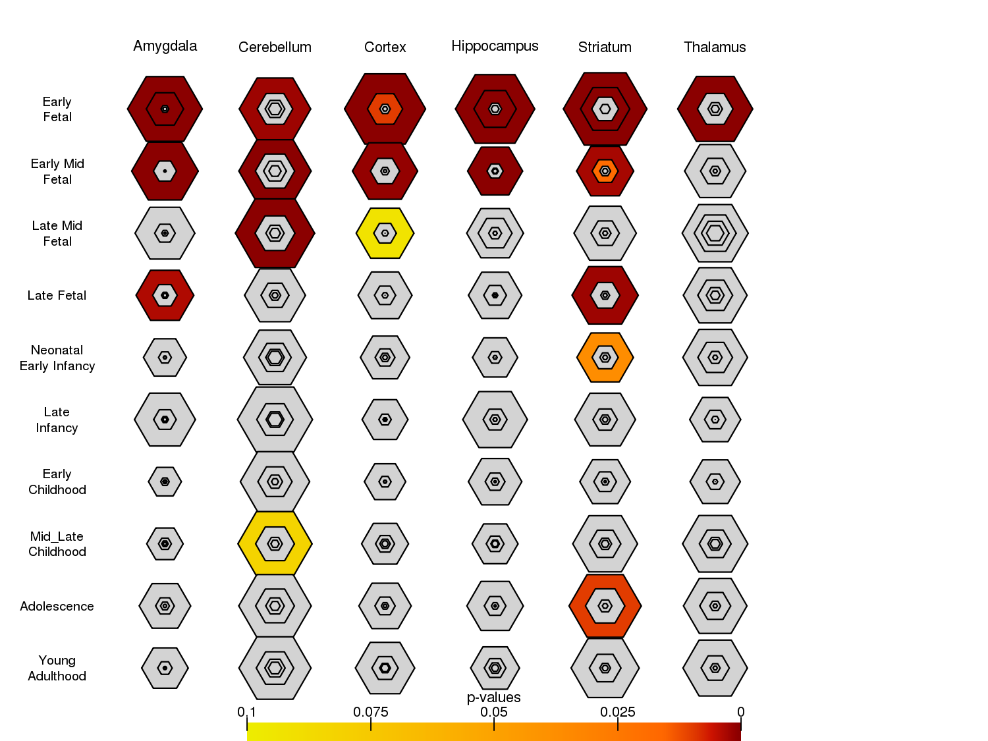
**Figure S5.** Characterization of 904 unique gene targets of 94 miRNAs found to be downregulated in mouse prefrontal cortex or hippocampus due to *DGCR8* deficiency and validated with strong experimental evidence in miRTarBase v7.0. A) Top five significantly enriched biological process, molecular function, and cellular component gene ontology (GO) terms; B) enrichment for specific developmental periods and brain regions; and C) enrichment for specific CNS cell types, defined at varying specificity indices using the Specific Expression Analysis tool [(Dougherty *et al.*, 2010)](https://paperpile.com/c/YtRzqs/vk47t). Varying specificity thresholds in (B) and (C) are represented by the hexagon ring layers going from the least specific gene lists (outer hexagons) to the most specific gene lists (center), with hexagons scaled to the size of gene lists. BH corrected Fisher’s Exact *p* values are plotted for each specificity threshold by color.

**B**

**A**


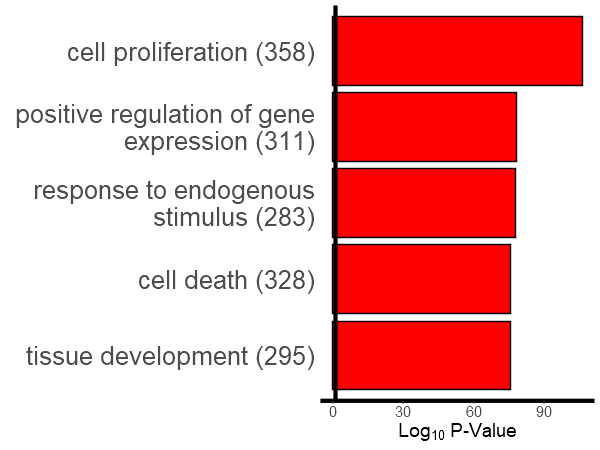


**C**


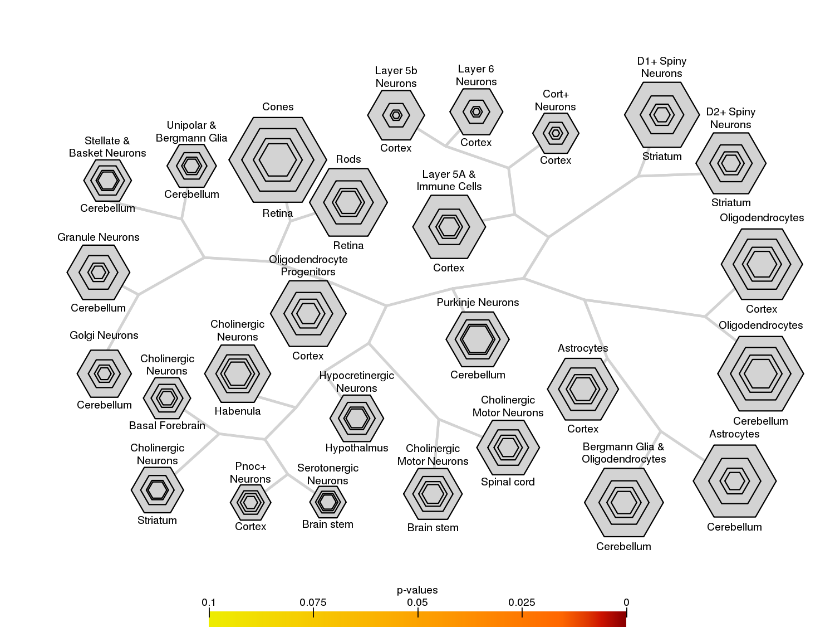


Table S1. Regional group differences in SA for 22q11DS patients and matched healthy controls (HC), adjusted for age, sex, and scanner. 22q11DS showed widespread reductions in SA compared to HC; SA deviance (ΔSA) Z-scores are calculated subtracting 22q11DS means from HC means and dividing the differences in group mean by the HC standard deviation (SD).

| Region | F value | Pr(>F) | FDR p | HC mean (SD) | 22q11DS mean (SD) | HC vs. 22q11DS ΔSA | 22q11DS ΔSA Z-Score |
| --- | --- | --- | --- | --- | --- | --- | --- |
| lh_bankssts | 39.89 | 5.78E-10 | **9.36E-10** | 981.28 (173.51) | 887.42 (162.53) | 93.86 | 0.54 |
| lh_caudalanteriorcingulate | 214.51 | 6.46E-41 | **4.39E-40** | 627.8 (125.83) | 487.36 (82.87) | 140.44 | 1.12 |
| lh_caudalmiddlefrontal | 20.28 | 8.28E-06 | **9.7E-06** | 2261.02 (396.98) | 2098.3 (426.29) | 162.73 | 0.41 |
| lh_cuneus | 360.80 | 1.69E-61 | **5.76E-60** | 1413.03 (196.53) | 1098.31 (176.99) | 314.72 | 1.60 |
| lh_entorhinal | 39.49 | 6.97E-10 | **1.08E-09** | 335.47 (76.88) | 293.62 (74.01) | 41.86 | 0.54 |
| lh_fusiform | 187.42 | 1.19E-36 | **5.77E-36** | 3198.6 (391.2) | 2714.94 (413.13) | 483.66 | 1.24 |
| lh_inferiorparietal | 26.65 | 3.48E-07 | **4.72E-07** | 4325.56 (604.88) | 4048.42 (615.26) | 277.14 | 0.46 |
| lh_inferiortemporal | 132.65 | 1.7E-27 | **5.78E-27** | 2881.38 (441.43) | 2407.97 (496.41) | 473.41 | 1.07 |
| lh_isthmuscingulate | 21.42 | 4.67E-06 | **5.88E-06** | 898.72 (162.01) | 832.87 (160.98) | 65.86 | 0.41 |
| lh_lateraloccipital | 158.51 | 6.49E-32 | **2.45E-31** | 4592.46 (557.87) | 3975.36 (554.68) | 617.10 | 1.11 |
| lh_lateralorbitofrontal | 31.58 | 3.12E-08 | **4.42E-08** | 2148.21 (299.9) | 1998.06 (307.57) | 150.15 | 0.50 |
| lh_lingual | 315.74 | 1.5E-55 | **2.55E-54** | 3036.16 (379.58) | 2442.35 (379.16) | 593.81 | 1.56 |
| lh_medialorbitofrontal | 21.23 | 5.13E-06 | **6.23E-06** | 1503.51 (244.82) | 1406.25 (233) | 97.26 | 0.40 |
| lh_middletemporal | 84.38 | 9.71E-19 | **2.54E-18** | 2763.24 (372.12) | 2441.76 (426.76) | 321.48 | 0.86 |
| lh_parahippocampal | 0.97 | 0.325866 | 0.346232 | 672.46 (100.71) | 663.91 (96.25) | 8.55 | 0.08 |
| lh_paracentral | 58.46 | 1.01E-13 | **1.81E-13** | 1277.3 (197.62) | 1149.3 (180.12) | 127.99 | 0.65 |
| lh_parsopercularis | 26.58 | 3.61E-07 | **4.72E-07** | 1708.55 (271.1) | 1587.91 (258.79) | 120.65 | 0.45 |
| lh_parsorbitalis | 32.07 | 2.46E-08 | **3.64E-08** | 589.23 (81.07) | 548.01 (84.55) | 41.22 | 0.51 |
| lh_parstriangularis | 81.21 | 3.87E-18 | **8.76E-18** | 1376.83 (192.32) | 1222.77 (196.27) | 154.06 | 0.80 |
| lh_pericalcarine | 234.18 | 6.5E-44 | **5.52E-43** | 1387.16 (225.07) | 1101.39 (194.42) | 285.77 | 1.27 |
| lh_postcentral | 131.37 | 2.84E-27 | **8.78E-27** | 4042.32 (485.77) | 3586.84 (403.68) | 455.48 | 0.94 |
| lh_posteriorcingulate | 62.54 | 1.57E-14 | **3.35E-14** | 1141.39 (160.94) | 1031.7 (153) | 109.69 | 0.68 |
| lh_precentral | 0.06 | 0.799853 | 0.799853 | 4486.01 (486.16) | 4474.28 (570.24) | 11.73 | 0.02 |
| lh_precuneus | 296.95 | 5.65E-53 | **6.4E-52** | 3698.12 (431.73) | 3064.5 (398.85) | 633.62 | 1.47 |
| lh_rostralanteriorcingulate | 110.69 | 1.34E-23 | **3.8E-23** | 675.52 (154.59) | 541.23 (131.81) | 134.29 | 0.87 |
| lh_rostralmiddlefrontal | 173.85 | 1.88E-34 | **7.99E-34** | 5762.14 (675.66) | 4977.04 (676.43) | 785.10 | 1.16 |
| lh_superiorfrontal | 60.70 | 3.63E-14 | **6.86E-14** | 6704.59 (773.24) | 6177.75 (760.68) | 526.85 | 0.68 |
| lh_superiorparietal | 205.15 | 1.84E-39 | **1.04E-38** | 5566.71 (659.58) | 4771.96 (590.76) | 794.74 | 1.20 |
| lh_superiortemporal | 62.10 | 1.92E-14 | **3.84E-14** | 3584.36 (411.59) | 3296.29 (419.25) | 288.08 | 0.70 |
| lh_supramarginal | 18.14 | 2.43E-05 | **2.67E-05** | 3704.31 (543.53) | 3497.84 (558.66) | 206.47 | 0.38 |
| lh_frontalpole | 19.33 | 1.33E-05 | **1.51E-05** | 205.1 (33.55) | 191.99 (34.25) | 13.12 | 0.39 |
| lh_temporalpole | 82.79 | 1.94E-18 | **4.71E-18** | 425.6 (62.22) | 376.49 (60.09) | 49.11 | 0.79 |
| lh_transversetemporal | 52.17 | 1.82E-12 | **3.1E-12** | 433.91 (70.69) | 387.06 (77.18) | 46.86 | 0.66 |
| lh_insula | 0.53 | 0.464984 | 0.479074 | 1835.47 (240.73) | 1818.75 (281.51) | 16.72 | 0.07 |

Table S2. Regional group differences in surface area (SA) for 22q11DS patients and healthy controls (HC), adjusted for age, sex, scanner, and ICV. 22q11DS showed widespread reductions in SA compared to HC; SA deviance (ΔSA) Z-scores are calculated subtracting 22q11DS means from HC means and dividing the differences in group mean by the HC standard deviation (SD).

| Region | F value | Pr(>F) | FDR p | HC mean (SD) | 22q11DS mean (SD) | HC vs. 22q11DS ΔSA | 22q11DS ΔSA Z-Score |
| --- | --- | --- | --- | --- | --- | --- | --- |
| lh_bankssts | 12.13 | 5.37E-04 | **7.94E-04** | 262.25 (159.07) | 214.45 (151.56) | 47.79 | 0.30 |
| lh_caudalanteriorcingulate | 137.57 | 2.38E-28 | **1.62E-27** | 88.88 (118.55) | -17.03 (77.93) | 105.91 | 0.89 |
| lh_caudalmiddlefrontal | 1.51 | 2.19E-01 | 2.33E-01 | 340.37 (364.07) | 300.7 (368.67) | 39.67 | 0.11 |
| lh_cuneus | 250.66 | 2.30E-46 | **7.84E-45** | 328.54 (186.77) | 83.31 (161.16) | 245.24 | 1.31 |
| lh_entorhinal | 13.13 | 3.19E-04 | **4.93E-04** | 36.23 (71.99) | 13.54 (69.92) | 22.69 | 0.32 |
| lh_fusiform | 106.31 | 8.34E-23 | **4.05E-22** | 727.83 (347.84) | 402.47 (370.89) | 325.37 | 0.94 |
| lh_inferiorparietal | 3.26 | 7.16E-02 | 8.40E-02 | 1327.96 (540.8) | 1242.87 (528.06) | 85.09 | 0.16 |
| lh_inferiortemporal | 68.22 | 1.22E-15 | **4.14E-15** | 127.82 (379.91) | -169.18 (441.08) | 297.00 | 0.78 |
| lh_isthmuscingulate | 2.40 | 1.22E-01 | 1.33E-01 | 183.1 (147.46) | 163.09 (145.29) | 20.01 | 0.14 |
| lh_lateraloccipital | 87.23 | 2.81E-19 | **1.06E-18** | 1174.64 (505.77) | 776.51 (455.19) | 398.13 | 0.79 |
| lh_lateralorbitofrontal | 3.29 | 7.04E-02 | 8.40E-02 | 436.75 (250.73) | 396.25 (257.08) | 40.50 | 0.16 |
| lh_lingual | 212.93 | 1.13E-40 | **1.93E-39** | 787.19 (347.7) | 337.47 (352.62) | 449.72 | 1.29 |
| lh_medialorbitofrontal | 1.31 | 2.53E-01 | 2.61E-01 | 314.11 (213.1) | 293.05 (203.89) | 21.06 | 0.10 |
| lh_middletemporal | 35.91 | 3.87E-09 | **8.77E-09** | 629.26 (329.24) | 444.5 (374.46) | 184.76 | 0.56 |
| lh_parahippocampal | 2.53 | 1.13E-01 | 1.28E-01 | 337.8 (93.28) | 350.69 (90.48) | -12.89 | -0.14 |
| lh_paracentral | 18.43 | 2.11E-05 | **3.77E-05** | 273.13 (176.68) | 209.47 (157.35) | 63.66 | 0.36 |
| lh_parsopercularis | 5.21 | 2.29E-02 | **2.89E-02** | 592.66 (250.88) | 543.51 (236.51) | 49.16 | 0.20 |
| lh_parsorbitalis | 8.37 | 3.97E-03 | **5.19E-03** | 250.3 (75.44) | 230.79 (77.86) | 19.51 | 0.26 |
| lh_parstriangularis | 40.78 | 3.79E-10 | **9.21E-10** | 599.57 (186.68) | 495.31 (183.72) | 104.26 | 0.56 |
| lh_pericalcarine | 156.60 | 1.36E-31 | **1.16E-30** | 469.12 (218.17) | 242.17 (189.42) | 226.95 | 1.04 |
| lh_postcentral | 66.07 | 3.21E-15 | **9.92E-15** | 1242.93 (428.88) | 966.8 (323.62) | 276.13 | 0.64 |
| lh_posteriorcingulate | 25.46 | 6.27E-07 | **1.33E-06** | 432.92 (151.58) | 368.62 (135.56) | 64.30 | 0.42 |
| lh_precentral | 16.42 | 5.84E-05 | **9.93E-05** | 1843.85 (417.33) | 2001.39 (469.65) | -157.54 | -0.38 |
| lh_precuneus | 203.40 | 3.47E-39 | **3.93E-38** | 953.1 (354.93) | 495.35 (375.9) | 457.76 | 1.29 |
| lh_rostralanteriorcingulate | 51.24 | 2.80E-12 | **7.93E-12** | -222.83 (129.56) | -299.56 (111.09) | 76.74 | 0.59 |
| lh_rostralmiddlefrontal | 97.43 | 3.53E-21 | **1.50E-20** | 1359.64 (571.87) | 856.6 (586.86) | 503.04 | 0.88 |
| lh_superiorfrontal | 15.91 | 7.61E-05 | **1.23E-04** | 1900.26 (624) | 1681.21 (622.91) | 219.05 | 0.35 |
| lh_superiorparietal | 123.26 | 7.59E-26 | **4.30E-25** | 1712.78 (571.44) | 1164.95 (545.83) | 547.83 | 0.96 |
| lh_superiortemporal | 18.88 | 1.67E-05 | **3.16E-05** | 1246.04 (353.94) | 1107.78 (370.17) | 138.27 | 0.39 |
| lh_supramarginal | 0.75 | 3.88E-01 | 3.88E-01 | 1058.69 (478.62) | 1021.72 (494.53) | 36.97 | 0.08 |
| lh_frontalpole | 8.42 | 3.86E-03 | **5.19E-03** | 133.59 (32.9) | 125.06 (33.97) | 8.53 | 0.26 |
| lh_temporalpole | 44.63 | 6.12E-11 | **1.60E-10** | 200.98 (59.04) | 166.26 (58.96) | 34.72 | 0.59 |
| lh_transversetemporal | 20.64 | 6.91E-06 | **1.38E-05** | 137.55 (66.42) | 109.68 (73.49) | 27.87 | 0.42 |
| lh_insula | 11.24 | 8.57E-04 | **1.21E-03** | 557.68 (208.08) | 622.82 (235.23) | -65.14 | -0.31 |

Table S3. Regional group differences in cortical thickness (CT) for 22q11DS patients and matched healthy controls (HC), adjusted for age, age^2^, sex, and scanner. 22q11DS showed increased CT in the majority of regions compared to HC; CT deviance (ΔCT) Z-scores are calculated subtracting HC means from 22q11DS means and dividing the differences in group means by the HC standard deviation (SD).

| Region | F value | Pr(>F) | FDR p | HC mean (SD) | 22q11DS mean (SD) | 22q11DS vs. HC ΔCT | 22q11DS ΔCT Z-Score |
| --- | --- | --- | --- | --- | --- | --- | --- |
| lh_bankssts | 0.70 | 4.02E-01 | 4.15E-01 | 3.23 (0.17) | 3.22 (0.2) | -0.01 | -0.08 |
| lh_caudalanteriorcingulate | 4.35 | 3.74E-02 | **4.71E-02** | 3.74 (0.24) | 3.69 (0.26) | -0.05 | -0.19 |
| lh_caudalmiddlefrontal | 44.05 | 8.07E-11 | **3.05E-10** | 2.98 (0.14) | 3.06 (0.15) | 0.08 | 0.61 |
| lh_cuneus | 30.76 | 4.66E-08 | **1.13E-07** | 2.5 (0.15) | 2.57 (0.15) | 0.07 | 0.48 |
| lh_entorhinal | 4.12 | 4.29E-02 | 5.21E-02 | 3.52 (0.35) | 3.59 (0.4) | 0.07 | 0.19 |
| lh_fusiform | 8.86 | 3.05E-03 | **4.32E-03** | 3.31 (0.14) | 3.34 (0.15) | 0.04 | 0.27 |
| lh_inferiorparietal | 6.36 | 1.20E-02 | **1.63E-02** | 3.13 (0.14) | 3.17 (0.15) | 0.03 | 0.23 |
| lh_inferiortemporal | 13.11 | 3.22E-04 | **5.22E-04** | 3.4 (0.17) | 3.46 (0.18) | 0.05 | 0.32 |
| lh_isthmuscingulate | 12.21 | 5.16E-04 | **7.98E-04** | 3.43 (0.19) | 3.48 (0.19) | 0.06 | 0.30 |
| lh_lateraloccipital | 5.96 | 1.49E-02 | **1.95E-02** | 2.73 (0.13) | 2.76 (0.13) | 0.03 | 0.21 |
| lh_lateralorbitofrontal | 31.84 | 2.75E-08 | **7.20E-08** | 3.41 (0.17) | 3.49 (0.18) | 0.09 | 0.51 |
| lh_lingual | 22.54 | 2.66E-06 | **5.32E-06** | 2.67 (0.13) | 2.73 (0.13) | 0.06 | 0.42 |
| lh_medialorbitofrontal | 47.40 | 1.68E-11 | **8.14E-11** | 3.28 (0.18) | 3.39 (0.19) | 0.11 | 0.62 |
| lh_middletemporal | 15.56 | 9.09E-05 | **1.54E-04** | 3.55 (0.18) | 3.61 (0.16) | 0.06 | 0.34 |
| lh_parahippocampal | 46.81 | 2.20E-11 | **9.37E-11** | 3.06 (0.3) | 2.89 (0.28) | -0.17 | -0.59 |
| lh_paracentral | 39.58 | 6.70E-10 | **2.28E-09** | 2.96 (0.16) | 3.05 (0.16) | 0.09 | 0.56 |
| lh_parsopercularis | 61.62 | 2.39E-14 | **2.71E-13** | 3.12 (0.14) | 3.22 (0.15) | 0.10 | 0.70 |
| lh_parsorbitalis | 24.14 | 1.20E-06 | **2.55E-06** | 3.43 (0.24) | 3.54 (0.24) | 0.10 | 0.44 |
| lh_parstriangularis | 34.39 | 8.04E-09 | **2.28E-08** | 3.13 (0.17) | 3.22 (0.18) | 0.09 | 0.52 |
| lh_pericalcarine | 67.28 | 1.86E-15 | **3.16E-14** | 1.94 (0.14) | 2.05 (0.16) | 0.11 | 0.77 |
| lh_postcentral | 56.50 | 2.48E-13 | **1.69E-12** | 2.55 (0.13) | 2.64 (0.15) | 0.09 | 0.71 |
| lh_posteriorcingulate | 1.57 | 2.10E-01 | 2.24E-01 | 3.48 (0.17) | 3.46 (0.15) | -0.02 | -0.10 |
| lh_precentral | 36.18 | 3.39E-09 | **1.05E-08** | 2.86 (0.13) | 2.93 (0.15) | 0.07 | 0.55 |
| lh_precuneus | 18.24 | 2.32E-05 | **4.15E-05** | 3.11 (0.13) | 3.16 (0.14) | 0.05 | 0.38 |
| lh_rostralanteriorcingulate | 2.88 | 9.03E-02 | 1.02E-01 | 3.75 (0.24) | 3.78 (0.28) | 0.04 | 0.16 |
| lh_rostralmiddlefrontal | 56.65 | 2.31E-13 | **1.69E-12** | 2.98 (0.14) | 3.08 (0.17) | 0.10 | 0.73 |
| lh_superiorfrontal | 30.60 | 5.04E-08 | **1.14E-07** | 3.49 (0.15) | 3.57 (0.15) | 0.07 | 0.50 |
| lh_superiorparietal | 9.63 | 2.02E-03 | **2.98E-03** | 2.63 (0.14) | 2.66 (0.13) | 0.04 | 0.27 |
| lh_superiortemporal | 22.01 | 3.47E-06 | **6.55E-06** | 3.34 (0.17) | 3.27 (0.16) | -0.07 | -0.40 |
| lh_supramarginal | 55.53 | 3.87E-13 | **2.19E-12** | 3.21 (0.15) | 3.31 (0.16) | 0.10 | 0.66 |
| lh_frontalpole | 2.31 | 1.29E-01 | 1.41E-01 | 3.66 (0.35) | 3.71 (0.37) | 0.05 | 0.14 |
| lh_temporalpole | 0.00 | 9.72E-01 | 9.72E-01 | 3.53 (0.36) | 3.53 (0.41) | 0.00 | 0.00 |
| lh_transversetemporal | 4.01 | 4.59E-02 | 5.38E-02 | 3.16 (0.24) | 3.12 (0.23) | -0.04 | -0.17 |
| lh_insula | 102.19 | 4.72E-22 | **1.60E-20** | 3.76 (0.17) | 3.9 (0.15) | 0.14 | 0.85 |

Table S4. Pearson’s *r* spatial correlations between expression of 22q11.2 genes and 22q11DS surface area deviance (ΔSA) severity, adjusted for age, sex, scanner, and ICV.

| ΔSA Spatial Association Rank | Gene | Pearson *r* | Bootstrap Pearson *r* Mean (SD) | Bootstrap 95% Confidence Interval | Bootstrap Pearson *r*  Z-Score | Pearson *r* Score AHBA Rank | Pearson *r* Z-Score *P*_AHBA_ |
| --- | --- | --- | --- | --- | --- | --- | --- |
| **1** | **DGCR8** | **0.50** | **0.48 (0.14)** | **0.18 - 0.70** | **3.52** | **0.991** | **0.009** |
| **2** | **AIFM3** | **0.40** | **0.39 (0.13)** | **0.11 - 0.62** | **2.97** | **0.971** | **0.029** |
| 3 | SCARF2 | 0.29 | 0.29 (0.16) | -0.03 - 0.56 | 1.87 | 0.862 | 0.138 |
| 4 | CLDN5 | 0.25 | 0.25 (0.16) | -0.08 - 0.54 | 1.58 | 0.818 | 0.182 |
| 5 | DGCR2 | 0.20 | 0.20 (0.15) | -0.09 - 0.50 | 1.31 | 0.773 | 0.227 |
| 6 | P2RX6 | 0.20 | 0.19 (0.17) | -0.17 - 0.50 | 1.13 | 0.741 | 0.259 |
| 7 | TANGO2 | 0.19 | 0.19 (0.18) | -0.16 - 0.53 | 1.06 | 0.727 | 0.273 |
| 8 | RANBP1 | 0.09 | 0.08 (0.12) | -0.16 - 0.32 | 0.68 | 0.656 | 0.344 |
| 9 | HIRA | 0.04 | 0.03 (0.15) | -0.25 - 0.32 | 0.22 | 0.566 | 0.434 |
| 10 | UFD1 | -0.01 | -0.01 (0.14) | -0.29 - 0.26 | -0.06 | 0.519 | 0.481 |
| 11 | ARVCF | -0.02 | -0.01 (0.17) | -0.34 - 0.31 | -0.08 | 0.515 | 0.485 |
| 12 | MED15 | -0.05 | -0.05 (0.17) | -0.38 - 0.29 | -0.28 | 0.480 | 0.520 |
| 13 | COMT | -0.04 | -0.05 (0.16) | -0.41 - 0.22 | -0.33 | 0.471 | 0.529 |
| 14 | PRODH | -0.11 | -0.11 (0.18) | -0.41 - 0.28 | -0.60 | 0.421 | 0.579 |
| 15 | SLC25A1 | -0.13 | -0.13 (0.18) | -0.47 - 0.24 | -0.74 | 0.395 | 0.605 |
| 16 | GNB1L | -0.11 | -0.11 (0.13) | -0.37 - 0.20 | -0.80 | 0.381 | 0.619 |
| 17 | GP1BB | -0.23 | -0.23 (0.14) | -0.49 - 0.03 | -1.68 | 0.210 | 0.790 |
| 18 | MRPL40 | -0.27 | -0.27 (0.16) | -0.55 - 0.06 | -1.71 | 0.204 | 0.796 |
| 19 | KLHL22 | -0.29 | -0.29 (0.15) | -0.55 - 0.04 | -1.85 | 0.180 | 0.820 |
| 20 | RIMBP3 | -0.37 | -0.35 (0.17) | -0.65 - 0.04 | -2.05 | 0.148 | 0.852 |
| 21 | PI4KA | -0.28 | -0.28 (0.13) | -0.53 - -0.02 | -2.09 | 0.141 | 0.859 |
| 22 | RTN4R | -0.32 | -0.33 (0.15) | -0.61 - -0.01 | -2.21 | 0.127 | 0.873 |
| 23 | C22orf39 | -0.40 | -0.38 (0.16) | -0.66 - -0.02 | -2.33 | 0.109 | 0.891 |
| 24 | SEPT5 | -0.36 | -0.36 (0.15) | -0.63 - -0.06 | -2.43 | 0.098 | 0.902 |
| 25 | DGCR6 | -0.32 | -0.32 (0.12) | -0.57 - -0.07 | -2.59 | 0.083 | 0.917 |
| 26 | SLC7A4 | -0.49 | -0.47 (0.16) | -0.72 - -0.10 | -3.04 | 0.048 | 0.952 |
| 27 | DGCR6L | -0.43 | -0.43 (0.12) | -0.65 - -0.18 | -3.48 | 0.026 | 0.974 |
| 28 | SNAP29 | -0.46 | -0.46 (0.12) | -0.66 - -0.20 | -3.88 | 0.013 | 0.987 |

Table S5. Spearman’s *ρ* spatial correlations between expression of 22q11.2 genes and 22q11DS surface area deviance (ΔSA) severity, adjusted for age, sex, and scanner.

| ΔSA Spatial Association Rank | Gene | Spearman *ρ* | Bootstrap Spearman *ρ* Mean (SD) | 95% Confidence Interval | Bootstrap Spearman *ρ* Z-Score | Spearman *ρ* Z-Score AHBA Rank | Spearman *ρ* Z-Score *P*_AHBA_ |
| --- | --- | --- | --- | --- | --- | --- | --- |
| **1** | **DGCR8** | **0.43** | **0.42 (0.16)** | **0.09 - 0.69** | **2.66** | **0.990** | **0.010** |
| **2** | **AIFM3** | **0.37** | **0.36 (0.17)** | **-0.01 - 0.64** | **2.18** | **0.963** | **0.037** |
| 3 | SCARF2 | 0.28 | 0.27 (0.18) | -0.14 - 0.59 | 1.48 | 0.870 | 0.130 |
| 4 | CLDN5 | 0.23 | 0.23 (0.17) | -0.13 - 0.53 | 1.32 | 0.837 | 0.163 |
| 5 | DGCR2 | 0.24 | 0.24 (0.18) | -0.15 - 0.57 | 1.31 | 0.834 | 0.166 |
| 6 | TANGO2 | 0.18 | 0.17 (0.18) | -0.19 - 0.51 | 0.96 | 0.755 | 0.245 |
| 7 | P2RX6 | 0.10 | 0.1 (0.19) | -0.28 - 0.44 | 0.55 | 0.664 | 0.336 |
| 8 | RANBP1 | 0.06 | 0.05 (0.17) | -0.27 - 0.37 | 0.32 | 0.610 | 0.390 |
| 9 | HIRA | 0.02 | 0.02 (0.19) | -0.33 - 0.37 | 0.11 | 0.561 | 0.439 |
| 10 | ARVCF | -0.01 | -0.01 (0.18) | -0.36 - 0.34 | -0.05 | 0.525 | 0.475 |
| 11 | PRODH | -0.03 | -0.03 (0.19) | -0.41 - 0.35 | -0.15 | 0.503 | 0.497 |
| 12 | GNB1L | -0.03 | -0.03 (0.17) | -0.33 - 0.31 | -0.20 | 0.492 | 0.508 |
| 13 | UFD1 | -0.04 | -0.04 (0.17) | -0.38 - 0.28 | -0.25 | 0.479 | 0.521 |
| 14 | MED15 | -0.06 | -0.06 (0.19) | -0.43 - 0.3 | -0.33 | 0.460 | 0.540 |
| 15 | COMT | -0.09 | -0.09 (0.21) | -0.46 - 0.34 | -0.42 | 0.439 | 0.561 |
| 16 | SLC25A1 | -0.13 | -0.14 (0.18) | -0.45 - 0.24 | -0.78 | 0.354 | 0.646 |
| 17 | RIMBP3 | -0.22 | -0.22 (0.19) | -0.57 - 0.13 | -1.19 | 0.256 | 0.744 |
| 18 | MRPL40 | -0.27 | -0.25 (0.18) | -0.59 - 0.14 | -1.38 | 0.210 | 0.790 |
| 19 | C22orf39 | -0.29 | -0.29 (0.18) | -0.61 - 0.1 | -1.59 | 0.162 | 0.838 |
| 20 | PI4KA | -0.28 | -0.27 (0.15) | -0.57 - 0.05 | -1.78 | 0.124 | 0.876 |
| 21 | GP1BB | -0.28 | -0.28 (0.16) | -0.57 - 0.03 | -1.82 | 0.117 | 0.883 |
| 22 | KLHL22 | -0.32 | -0.31 (0.17) | -0.62 - 0.05 | -1.86 | 0.110 | 0.890 |
| 23 | SLC7A4 | -0.37 | -0.35 (0.18) | -0.66 - 0.05 | -1.91 | 0.102 | 0.898 |
| 24 | SNAP29 | -0.33 | -0.33 (0.17) | -0.62 - 0.05 | -1.98 | 0.091 | 0.909 |
| 25 | SEPT5 | -0.35 | -0.34 (0.17) | -0.65 - 0.04 | -2.03 | 0.084 | 0.916 |
| 26 | DGCR6 | -0.35 | -0.34 (0.16) | -0.61 0 | -2.21 | 0.064 | 0.936 |
| 27 | RTN4R | -0.44 | -0.43 (0.16) | -0.73 - -0.07 | -2.68 | 0.029 | 0.971 |
| 28 | DGCR6L | -0.42 | -0.41 (0.15) | -0.67 - -0.07 | -2.71 | 0.027 | 0.973 |

Table S6. Spearman’s *ρ* spatial correlations between expression of 22q11.2 genes and 22q11DS cortical thickness deviance (ΔCT) severity, adjusted for age, age^2^, sex, and scanner.

| ΔCT Spatial Association Rank | Gene | Spearman *ρ* | Bootstrap Spearman *ρ* Mean (SD) | 95% Confidence Interval | Bootstrap Spearman *ρ* Z-Score | Spearman *ρ* Z-Score AHBA Rank | Spearman *ρ* Z-Score *P*_AHBA_ |
| --- | --- | --- | --- | --- | --- | --- | --- |
| **1** | **GNB1L** | **0.38** | **0.38 (0.15)** | **0.07 - 0.64** | **2.65** | **0.961** | **0.039** |
| **2** | **P2RX6** | **0.39** | **0.38 (0.15)** | **0.07 - 0.63** | **2.6** | **0.958** | **0.042** |
| 3 | TANGO2 | 0.29 | 0.28 (0.17) | -0.06 - 0.59 | 1.69 | 0.874 | 0.126 |
| 4 | MRPL40 | 0.25 | 0.24 (0.17) | -0.1 - 0.56 | 1.42 | 0.831 | 0.169 |
| 5 | *DGCR8* | 0.24 | 0.24 (0.18) | -0.14 - 0.55 | 1.32 | 0.812 | 0.188 |
| 6 | CLDN5 | 0.19 | 0.19 (0.16) | -0.13 - 0.48 | 1.24 | 0.796 | 0.204 |
| 7 | SCARF2 | 0.21 | 0.21 (0.18) | -0.15 - 0.55 | 1.15 | 0.781 | 0.219 |
| 8 | RANBP1 | 0.20 | 0.19 (0.17) | -0.16 - 0.51 | 1.13 | 0.777 | 0.223 |
| 9 | AIFM3 | 0.20 | 0.19 (0.19) | -0.18 - 0.56 | 0.99 | 0.749 | 0.251 |
| 10 | DGCR2 | 0.19 | 0.18 (0.18) | -0.2 - 0.52 | 0.98 | 0.746 | 0.254 |
| 11 | KLHL22 | 0.15 | 0.15 (0.19) | -0.26 - 0.53 | 0.78 | 0.706 | 0.294 |
| 12 | RIMBP3 | 0.13 | 0.14 (0.19) | -0.24 - 0.51 | 0.72 | 0.692 | 0.308 |
| 13 | RTN4R | 0.13 | 0.12 (0.19) | -0.27 - 0.48 | 0.64 | 0.672 | 0.328 |
| 14 | UFD1 | 0.11 | 0.1 (0.19) | -0.28 - 0.45 | 0.54 | 0.648 | 0.352 |
| 15 | HIRA | 0.09 | 0.09 (0.18) | -0.26 - 0.44 | 0.49 | 0.634 | 0.366 |
| 16 | MED15 | 0.05 | 0.06 (0.17) | -0.28 - 0.42 | 0.33 | 0.589 | 0.411 |
| 17 | PI4KA | 0.01 | 0.01 (0.18) | -0.35 - 0.36 | 0.06 | 0.512 | 0.488 |
| 18 | COMT | 0.01 | 0.01 (0.18) | -0.33 - 0.38 | 0.06 | 0.512 | 0.488 |
| 19 | DGCR6L | -0.04 | -0.03 (0.18) | -0.37 - 0.32 | -0.18 | 0.446 | 0.554 |
| 20 | ARVCF | -0.03 | -0.03 (0.18) | -0.38 - 0.35 | -0.18 | 0.445 | 0.555 |
| 21 | DGCR6 | -0.11 | -0.11 (0.17) | -0.43 - 0.23 | -0.65 | 0.326 | 0.674 |
| 22 | SEPT5 | -0.12 | -0.12 (0.17) | -0.43 - 0.23 | -0.7 | 0.311 | 0.689 |
| 23 | C22orf39 | -0.21 | -0.2 (0.18) | -0.53 - 0.19 | -1.09 | 0.228 | 0.772 |
| 24 | SLC7A4 | -0.28 | -0.29 (0.18) | -0.64 - 0.08 | -1.59 | 0.132 | 0.868 |
| 25 | PRODH | -0.27 | -0.28 (0.17) | -0.59 - 0.08 | -1.62 | 0.125 | 0.875 |
| 26 | SLC25A1 | -0.27 | -0.27 (0.16) | -0.56 - 0.07 | -1.68 | 0.114 | 0.886 |
| 27 | GP1BB | -0.30 | -0.3 (0.17) | -0.62 - 0.05 | -1.75 | 0.107 | 0.893 |
| 28 | SNAP29 | -0.34 | -0.33 (0.15) | -0.62 - -0.01 | -2.14 | 0.058 | 0.942 |

Table S7. Spatial correlations between expression of consistently expressed 22q11.2 genes and 22q11DS ΔSA severity, adjusted for age, sex, and scanner with Z-score percentile ranks and *P*_AHBA_ values shown relative to the empirical distribution of all 4947 brain-expressed, protein-coding genes with consistent expression levels across the 6 AHBA donors (defined as average donor-to-median expression, *ρ* > 0.446, French and Paus, 2015).

| Gene | Average Donor-to-Median Consistency | Pearson *r* | Bootstrap Pearson *r* Mean (SD) | Bootstrap 95% Confidence Interval | Bootstrap Pearson *r*  Z-Score | Pearson *r* Z-Score AHBA Rank | Pearson *r* Z-Score *P*_AHBA_ |
| --- | --- | --- | --- | --- | --- | --- | --- |
| **DGCR8** | **0.703** | **0.53** | **0.52 (0.13)** | **0.21 - 0.72** | **4.03** | **0.991** | **0.009** |
| AIFM3 | 0.605 | 0.42 | 0.41 (0.14) | 0.11 - 0.64 | 2.95 | 0.935 | 0.065 |
| SCARF2 | 0.363 | 0.30 | 0.30 (0.17) | -0.07 - 0.6 | 1.78 | *NA* | *NA* |
| CLDN5 | 0.535 | 0.27 | 0.26 (0.16) | -0.06 - 0.54 | 1.62 | 0.739 | 0.261 |
| DGCR2 | 0.440 | 0.23 | 0.23 (0.15) | -0.07 - 0.51 | 1.56 | *NA* | *NA* |
| P2RX6 | 0.822 | 0.22 | 0.22 (0.15) | -0.10 - 0.53 | 1.44 | 0.707 | 0.293 |
| TANGO2 | 0.593 | 0.22 | 0.22 (0.17) | -0.13 - 0.53 | 1.31 | 0.688 | 0.312 |
| RANBP1 | 0.215 | 0.13 | 0.13 (0.14) | -0.16 - 0.37 | 0.92 | *NA* | *NA* |
| HIRA | 0.370 | 0.05 | 0.06 (0.16) | -0.28 - 0.36 | 0.36 | *NA* | *NA* |
| UFD1 | 0.476 | 0.03 | 0.02 (0.15) | -0.28 - 0.29 | 0.14 | 0.544 | 0.456 |
| ARVCF | 0.481 | 0 | 0 (0.18) | -0.38 - 0.35 | 0.02 | 0.537 | 0.463 |
| COMT | 0.503 | 0 | -0.02 (0.19) | -0.44 - 0.28 | -0.12 | 0.523 | 0.477 |
| MED15 | 0.361 | -0.05 | -0.06 (0.17) | -0.39 - 0.31 | -0.33 | *NA* | *NA* |
| GNB1L | 0.434 | -0.07 | -0.06 (0.15) | -0.34 - 0.24 | -0.42 | *NA* | *NA* |
| PRODH | 0.483 | -0.11 | -0.11 (0.18) | -0.45 - 0.26 | -0.62 | 0.472 | 0.528 |
| SLC25A1 | 0.428 | -0.15 | -0.16 (0.18) | -0.50 - 0.20 | -0.88 | *NA* | *NA* |
| MRPL40 | 0.306 | -0.28 | -0.28 (0.17) | -0.59 - 0.07 | -1.70 | *NA* | *NA* |
| GP1BB | 0.684 | -0.25 | -0.25 (0.13) | -0.50 - 0.04 | -1.84 | 0.269 | 0.731 |
| PI4KA | 0.479 | -0.26 | -0.26 (0.14) | -0.50 - 0.02 | -1.85 | 0.267 | 0.733 |
| RIMBP3 | 0.550 | -0.33 | -0.32 (0.17) | -0.62 - 0.04 | -1.88 | 0.260 | 0.740 |
| KLHL22 | 0.227 | -0.30 | -0.29 (0.15) | -0.58 - 0.02 | -1.96 | *NA* | *NA* |
| RTN4R | 0.439 | -0.32 | -0.31 (0.15) | -0.59 - -0.02 | -2.09 | *NA* | *NA* |
| C22orf39 | 0.717 | -0.40 | -0.38 (0.17) | -0.67 - 0.04 | -2.20 | 0.199 | 0.801 |
| SEPT5 | 0.594 | -0.39 | -0.39 (0.15) | -0.67 - -0.05 | -2.51 | 0.854 | 0.139 |
| DGCR6 | 0.357 | -0.36 | -0.36 (0.12) | -0.59 - -0.10 | -2.89 | *NA* | *NA* |
| DGCR6L | 0.425 | -0.45 | -0.45 (0.13) | -0.68 - -0.16 | -3.37 | *NA* | *NA* |
| SNAP29 | 0.551 | -0.47 | -0.46 (0.12) | -0.67 - -0.19 | -3.76 | 0.027 | 0.973 |
| SLC7A4 | 0.773 | -0.51 | -0.51 (0.13) | -0.74 - -0.21 | -3.82 | 0.026 | 0.974 |

Table S8. Spatial correlations between expression of consistently expressed 22q11.2 genes and 22q11DS ΔCT severity, adjusted for age, sex, and scanner with Z-score *P*_AHBA_ values shown relative to the empirical distribution of all 4947 brain-expressed, protein-coding genes with consistent expression levels across the 6 AHBA donors (defined as average donor-to-median expression, *ρ* > 0.446, French and Paus, 2015).

| Gene | Average Donor-to-Median Consistency | Pearson *r* | Boostrap Pearson *r* Mean (SD) | 95% Confidence Interval | Bootstrap Pearson *r* Z-Score | Pearson *r* Z-Score AHBA Rank | Pearson *r* Z-Score *P*_AHBA_ |
| --- | --- | --- | --- | --- | --- | --- | --- |
| **P2RX6** | **0.822** | **0.43** | **0.42 (0.12)** | **0.15 - 0.64** | **3.46** | **0.967** | **0.033** |
| GNB1L | 0.434 | 0.34 | 0.35 (0.13) | 0.09 - 0.61 | 2.62 | *NA* | *NA* |
| RANBP1 | 0.215 | 0.23 | 0.23 (0.14) | -0.07 - 0.49 | 1.62 | *NA* | *NA* |
| AIFM3 | 0.605 | 0.28 | 0.27 (0.17) | -0.10 - 0.58 | 1.54 | 0.752 | 0.248 |
| TANGO2 | 0.593 | 0.25 | 0.25 (0.17) | -0.08 - 0.56 | 1.50 | 0.746 | 0.254 |
| SCARF2 | 0.363 | 0.24 | 0.23 (0.17) | -0.14 - 0.51 | 1.37 | *NA* | *NA* |
| DGCR8 | 0.703 | 0.24 | 0.22 (0.16) | -0.11 - 0.5 | 1.36 | 0.724 | 0.276 |
| CLDN5 | 0.535 | 0.19 | 0.18 (0.14) | -0.10 - 0.46 | 1.30 | 0.714 | 0.286 |
| MRPL40 | 0.306 | 0.17 | 0.17 (0.14) | -0.10 - 0.42 | 1.22 | *NA* | *NA* |
| DGCR2 | 0.440 | 0.17 | 0.18 (0.18) | -0.19 - 0.50 | 1 | *NA* | *NA* |
| MED15 | 0.361 | 0.15 | 0.14 (0.17) | -0.18 - 0.46 | 0.86 | *NA* | *NA* |
| HIRA | 0.370 | 0.11 | 0.10 (0.15) | -0.19 - 0.38 | 0.67 | *NA* | *NA* |
| KLHL22 | 0.227 | 0.09 | 0.10 (0.19) | -0.27 - 0.47 | 0.52 | *NA* | *NA* |
| COMT | 0.439 | 0.04 | 0.04 (0.15) | -0.30 - 0.30 | 0.25 | 0.540 | 0.460 |
| RTN4R | 0.503 | 0.05 | 0.05 (0.2) | -0.3 - 0.45 | 0.25 | *NA* | *NA* |
| UFD1 | 0.476 | 0.01 | 0.01 (0.17) | -0.33 - 0.34 | 0.07 | 0.509 | 0.491 |
| ARVCF | 0.481 | 0.01 | 0.01 (0.16) | -0.31 - 0.33 | 0.05 | 0.503 | 0.497 |
| RIMBP3 | 0.550 | -0.01 | 0 (0.18) | -0.33 - 0.38 | 0.01 | 0.497 | 0.503 |
| PI4KA | 0.479 | -0.01 | -0.01 (0.16) | -0.33 - 0.31 | -0.08 | 0.483 | 0.517 |
| DGCR6L | 0.425 | -0.10 | -0.09 (0.17) | -0.42 - 0.25 | -0.52 | *NA* | *NA* |
| SEPT5 | 0.594 | -0.15 | -0.14 (0.17) | -0.44 - 0.19 | -0.82 | 0.356 | 0.644 |
| DGCR6 | 0.357 | -0.20 | -0.18 (0.15) | -0.45 - 0.14 | -1.22 | *NA* | *NA* |
| GP1BB | 0.684 | -0.22 | -0.24 (0.16) | -0.56 - 0.08 | -1.46 | 0.246 | 0.754 |
| PRODH | 0.483 | -0.27 | -0.26 (0.17) | -0.55 - 0.11 | -1.49 | 0.241 | 0.759 |
| C22orf39 | 0.717 | -0.28 | -0.26 (0.16) | -0.54 - 0.09 | -1.61 | 0.216 | 0.784 |
| SLC7A4 | 0.773 | -0.31 | -0.30 (0.16) | -0.57 - 0.04 | -1.90 | 0.167 | 0.833 |
| SLC25A1 | 0.551 | -0.36 | -0.36 (0.16) | -0.64 - 0 | -2.26 | *NA* | *NA* |
| SNAP29 | 0.428 | -0.34 | -0.33 (0.12) | -0.53 - -0.08 | -2.86 | 0.049 | 0.951 |

Table S9. Z-score 22q11DS surface area deviation (ΔSA) compared to healthy controls (HC) for children, adolescents, and adults, adjusted for age, sex, and scanner.

|  | Childhood (≤12 y) | | | | Adolescence (13-17 y) | | | | Adult (≥18 y) | | | |
| --- | --- | --- | --- | --- | --- | --- | --- | --- | --- | --- | --- | --- |
| Region | F | Pr(>F) | FDR p | HC vs. 22q11DS ΔSA Z-Score | F | Pr(>F) | FDR p | HC vs. 22q11DS ΔSA Z-Score | F | Pr(>F) | FDR p | HC vs. 22q11DS ΔSA Z-Score |
| lh_bankssts | 8.71 | 3.63E-03 | **4.93E-03** | 0.43 | 12.04 | 6.90E-04 | **1.23E-03** | 0.60 | 19.68 | 1.47E-05 | **2.38E-05** | 0.59 |
| lh_caudalanteriorcingulate | 79.47 | 9.82E-16 | **8.66E-15** | 1.24 | 71.38 | 3.16E-14 | **3.41E-13** | 1.17 | 69.90 | 8.24E-15 | **3.50E-14** | 1.01 |
| lh_caudalmiddlefrontal | 6.53 | 1.15E-02 | **1.30E-02** | 0.40 | 3.11 | 8.02E-02 | 9.08E-02 | 0.30 | 11.77 | 7.25E-04 | **9.86E-04** | 0.50 |
| lh_cuneus | 87.59 | 6.52E-17 | **1.11E-15** | 1.38 | 108.95 | 2.78E-19 | **9.46E-18** | 1.72 | 168.60 | 9.42E-29 | **3.20E-27** | 1.71 |
| lh_entorhinal | 7.28 | 7.71E-03 | **9.71E-03** | 0.41 | 12.40 | 5.78E-04 | **1.16E-03** | 0.59 | 20.79 | 8.67E-06 | **1.47E-05** | 0.62 |
| lh_fusiform | 67.76 | 5.80E-14 | **2.82E-13** | 1.21 | 40.80 | 2.25E-09 | **9.57E-09** | 1.16 | 78.00 | 3.95E-16 | **1.92E-15** | 1.32 |
| lh_inferiorparietal | 7.21 | 8.02E-03 | **9.74E-03** | 0.46 | 11.51 | 8.99E-04 | **1.53E-03** | 0.58 | 9.01 | 3.00E-03 | **3.65E-03** | 0.40 |
| lh_inferiortemporal | 47.82 | 1.03E-10 | **3.17E-10** | 1.21 | 24.56 | 2.02E-06 | **6.26E-06** | 0.97 | 64.06 | 7.83E-14 | **2.96E-13** | 1.06 |
| lh_isthmuscingulate | 23.52 | 2.87E-06 | **5.43E-06** | 0.70 | 1.44 | 2.32E-01 | 2.47E-01 | 0.21 | 4.33 | 3.88E-02 | **4.25E-02** | 0.29 |
| lh_lateraloccipital | 56.23 | 4.01E-12 | **1.70E-11** | 1.23 | 41.98 | 1.41E-09 | **6.86E-09** | 1.03 | 60.01 | 3.85E-13 | **1.31E-12** | 1.07 |
| lh_lateralorbitofrontal | 14.49 | 1.99E-04 | **2.83E-04** | 0.59 | 5.41 | 2.15E-02 | **2.92E-02** | 0.42 | 12.01 | 6.39E-04 | **9.06E-04** | 0.47 |
| lh_lingual | 99.65 | 1.37E-18 | **4.66E-17** | 1.52 | 93.94 | 2.31E-17 | **3.93E-16** | 1.61 | 122.47 | 9.19E-23 | **1.04E-21** | 1.57 |
| lh_medialorbitofrontal | 6.79 | 1.00E-02 | **1.17E-02** | 0.40 | 3.36 | 6.90E-02 | 8.38E-02 | 0.31 | 11.31 | 9.13E-04 | **1.19E-03** | 0.44 |
| lh_middletemporal | 37.26 | 7.37E-09 | **1.79E-08** | 1.00 | 18.25 | 3.53E-05 | **8.58E-05** | 0.87 | 31.64 | 5.82E-08 | **1.32E-07** | 0.78 |
| lh_parahippocampal | 0.11 | 7.37E-01 | 7.59E-01 | 0.05 | 0.32 | 5.74E-01 | 5.74E-01 | -0.09 | 2.60 | 1.08E-01 | 1.15E-01 | 0.22 |
| lh_paracentral | 25.92 | 9.80E-07 | **2.08E-06** | 0.75 | 6.19 | 1.40E-02 | **1.99E-02** | 0.42 | 29.71 | 1.39E-07 | **2.78E-07** | 0.72 |
| lh_parsopercularis | 20.88 | 9.65E-06 | **1.56E-05** | 0.65 | 3.21 | 7.51E-02 | 8.81E-02 | 0.30 | 6.27 | 1.30E-02 | **1.48E-02** | 0.35 |
| lh_parsorbitalis | 15.89 | 1.01E-04 | **1.50E-04** | 0.59 | 3.62 | 5.90E-02 | 7.52E-02 | 0.35 | 14.20 | 2.13E-04 | **3.15E-04** | 0.54 |
| lh_parstriangularis | 43.04 | 6.89E-10 | **1.80E-09** | 0.96 | 9.58 | 2.37E-03 | **3.83E-03** | 0.54 | 33.34 | 2.72E-08 | **6.61E-08** | 0.83 |
| lh_pericalcarine | 72.76 | 9.91E-15 | **5.61E-14** | 1.20 | 51.15 | 4.16E-11 | **2.83E-10** | 1.17 | 112.09 | 2.67E-21 | **2.27E-20** | 1.39 |
| lh_postcentral | 54.24 | 8.52E-12 | **2.90E-11** | 1.06 | 24.69 | 1.91E-06 | **6.26E-06** | 0.81 | 54.41 | 3.62E-12 | **1.12E-11** | 0.94 |
| lh_posteriorcingulate | 33.95 | 2.96E-08 | **6.72E-08** | 0.96 | 12.60 | 5.23E-04 | **1.11E-03** | 0.57 | 18.92 | 2.12E-05 | **3.27E-05** | 0.58 |
| lh_precentral | 0.05 | 8.29E-01 | 8.29E-01 | 0.04 | 0.35 | 5.56E-01 | 5.73E-01 | -0.10 | 0.56 | 4.54E-01 | 4.54E-01 | 0.11 |
| lh_precuneus | 78.33 | 1.45E-15 | **9.84E-15** | 1.36 | 70.67 | 4.01E-14 | **3.41E-13** | 1.40 | 156.16 | 3.26E-27 | **5.54E-26** | 1.60 |
| lh_rostralanteriorcingulate | 43.81 | 5.06E-10 | **1.43E-09** | 0.99 | 21.62 | 7.53E-06 | **1.97E-05** | 0.71 | 45.60 | 1.37E-10 | **3.88E-10** | 0.87 |
| lh_rostralmiddlefrontal | 55.50 | 5.28E-12 | **2.00E-11** | 1.12 | 35.30 | 2.08E-08 | **7.87E-08** | 1.04 | 85.03 | 3.02E-17 | **1.71E-16** | 1.27 |
| lh_superiorfrontal | 24.81 | 1.60E-06 | **3.21E-06** | 0.77 | 12.25 | 6.21E-04 | **1.17E-03** | 0.62 | 24.10 | 1.83E-06 | **3.45E-06** | 0.65 |
| lh_superiorparietal | 79.36 | 1.02E-15 | **8.66E-15** | 1.26 | 42.31 | 1.24E-09 | **6.86E-09** | 1.05 | 86.21 | 1.97E-17 | **1.34E-16** | 1.26 |
| lh_superiortemporal | 16.98 | 6.00E-05 | **9.27E-05** | 0.63 | 15.10 | 1.56E-04 | **3.54E-04** | 0.73 | 30.92 | 8.04E-08 | **1.71E-07** | 0.75 |
| lh_supramarginal | 8.15 | 4.86E-03 | **6.35E-03** | 0.46 | 2.43 | 1.21E-01 | 1.33E-01 | 0.27 | 7.84 | 5.57E-03 | **6.53E-03** | 0.39 |
| lh_frontalpole | 3.76 | 5.41E-02 | 5.93E-02 | 0.34 | 7.09 | 8.66E-03 | **1.28E-02** | 0.45 | 10.00 | 1.79E-03 | **2.26E-03** | 0.40 |
| lh_temporalpole | 23.32 | 3.16E-06 | **5.65E-06** | 0.72 | 23.06 | 3.93E-06 | **1.11E-05** | 0.80 | 36.06 | 8.21E-09 | **2.15E-08** | 0.84 |
| lh_transversetemporal | 21.07 | 8.85E-06 | **1.50E-05** | 0.76 | 9.30 | 2.74E-03 | **4.24E-03** | 0.56 | 22.54 | 3.78E-06 | **6.77E-06** | 0.66 |
| lh_insula | 2.01 | 1.58E-01 | 1.68E-01 | 0.23 | 3.60 | 5.98E-02 | 7.52E-02 | -0.39 | 1.52 | 2.19E-01 | 2.26E-01 | 0.18 |

Table S10. Z-score 22q11DS cortical thickness deviation (ΔCT) compared to healthy controls (HC) for children, adolescents, and adults, adjusted for age, age^2^, sex, and scanner.

|  | Childhood (≤12 y) | | | | Adolescence (13-17 y) | | | | Adult (≥18 y) | | | |
| --- | --- | --- | --- | --- | --- | --- | --- | --- | --- | --- | --- | --- |
| Region | F value | Pr(>F) | FDR p | 22q11DS vs. HC ΔCT  Z-Score | F value | Pr(>F) | FDR p | 22q11DS vs. HC ΔCT  Z-Score | F value | Pr(>F) | FDR p | 22q11DS vs. HC ΔCT  Z-Score |
| lh_bankssts | 0.51 | 4.74E-01 | 5.56E-01 | -0.12 | 1.37 | 2.43E-01 | 3.31E-01 | -0.22 | 0.16 | 6.88E-01 | 6.88E-01 | 0.06 |
| lh_caudalanteriorcingulate | 1.35 | 2.47E-01 | 3.11E-01 | -0.20 | 1.17 | 2.81E-01 | 3.67E-01 | -0.19 | 1.74 | 1.88E-01 | 2.13E-01 | -0.18 |
| lh_caudalmiddlefrontal | 24.65 | 1.72E-06 | **2.93E-05** | 0.91 | 3.44 | 6.57E-02 | 1.18E-01 | 0.32 | 19.51 | 1.60E-05 | **5.85E-05** | 0.59 |
| lh_cuneus | 3.31 | 7.07E-02 | 1.05E-01 | 0.30 | 2.46 | 1.19E-01 | 1.84E-01 | 0.25 | 37.69 | 4.03E-09 | **4.57E-08** | 0.80 |
| lh_entorhinal | 0.20 | 6.57E-01 | 7.20E-01 | 0.08 | 0.10 | 7.54E-01 | 8.20E-01 | 0.05 | 7.33 | 7.35E-03 | **1.09E-02** | 0.38 |
| lh_fusiform | 8.26 | 4.60E-03 | **9.78E-03** | 0.47 | 0.08 | 7.71E-01 | 8.20E-01 | 0.05 | 4.09 | 4.45E-02 | 5.82E-02 | 0.27 |
| lh_inferiorparietal | 0.57 | 4.53E-01 | 5.50E-01 | 0.13 | 0.65 | 4.22E-01 | 5.32E-01 | 0.15 | 7.85 | 5.55E-03 | **8.99E-03** | 0.35 |
| lh_inferiortemporal | 11.37 | 9.34E-04 | **2.64E-03** | 0.56 | 1.38 | 2.43E-01 | 3.31E-01 | 0.21 | 3.00 | 8.50E-02 | 1.03E-01 | 0.22 |
| lh_isthmuscingulate | 10.63 | 1.36E-03 | **3.55E-03** | 0.51 | 3.26 | 7.33E-02 | 1.25E-01 | 0.30 | 1.11 | 2.92E-01 | 3.11E-01 | 0.14 |
| lh_lateraloccipital | 1.80 | 1.82E-01 | 2.48E-01 | 0.24 | 0.26 | 6.13E-01 | 6.95E-01 | 0.09 | 4.97 | 2.69E-02 | **3.81E-02** | 0.28 |
| lh_lateralorbitofrontal | 13.13 | 3.89E-04 | **1.32E-03** | 0.60 | 4.36 | 3.87E-02 | 7.99E-02 | 0.39 | 15.67 | 1.03E-04 | **2.91E-04** | 0.52 |
| lh_lingual | 6.47 | 1.19E-02 | **2.13E-02** | 0.41 | 4.30 | 4.00E-02 | 7.99E-02 | 0.35 | 12.79 | 4.32E-04 | **8.63E-04** | 0.49 |
| lh_medialorbitofrontal | 22.26 | 5.10E-06 | **4.34E-05** | 0.85 | 7.73 | 6.18E-03 | **2.01E-02** | 0.45 | 18.47 | 2.63E-05 | **8.13E-05** | 0.56 |
| lh_middletemporal | 5.46 | 2.07E-02 | **3.52E-02** | 0.34 | 2.79 | 9.73E-02 | 1.58E-01 | 0.31 | 7.69 | 6.04E-03 | **9.33E-03** | 0.35 |
| lh_parahippocampal | 12.57 | 5.14E-04 | **1.59E-03** | -0.56 | 20.88 | 1.05E-05 | **1.79E-04** | -0.80 | 14.71 | 1.65E-04 | **3.74E-04** | -0.49 |
| lh_paracentral | 14.93 | 1.61E-04 | **6.85E-04** | 0.64 | 5.46 | 2.09E-02 | **4.73E-02** | 0.44 | 20.29 | 1.10E-05 | **4.66E-05** | 0.58 |
| lh_parsopercularis | 23.29 | 3.20E-06 | **3.62E-05** | 0.76 | 14.85 | 1.76E-04 | **1.60E-03** | 0.68 | 23.69 | 2.21E-06 | **1.50E-05** | 0.67 |
| lh_parsorbitalis | 7.51 | 6.83E-03 | **1.29E-02** | 0.42 | 7.58 | 6.69E-03 | **2.01E-02** | 0.48 | 8.92 | 3.16E-03 | **5.36E-03** | 0.42 |
| lh_parstriangularis | 18.53 | 2.89E-05 | **1.64E-04** | 0.67 | 7.46 | 7.10E-03 | **2.01E-02** | 0.50 | 9.82 | 1.97E-03 | **3.52E-03** | 0.42 |
| lh_pericalcarine | 9.18 | 2.85E-03 | **6.91E-03** | 0.47 | 14.16 | 2.45E-04 | **1.67E-03** | 0.69 | 60.24 | 3.51E-13 | **1.19E-11** | 1.21 |
| lh_postcentral | 13.27 | 3.62E-04 | **1.32E-03** | 0.61 | 9.73 | 2.20E-03 | **1.07E-02** | 0.57 | 38.91 | 2.37E-09 | **4.03E-08** | 0.89 |
| lh_posteriorcingulate | 0.06 | 8.15E-01 | 8.39E-01 | 0.04 | 0.01 | 9.06E-01 | 9.06E-01 | 0.02 | 4.43 | 3.65E-02 | **4.97E-02** | -0.26 |
| lh_precentral | 8.75 | 3.55E-03 | **8.05E-03** | 0.48 | 8.56 | 4.00E-03 | **1.70E-02** | 0.55 | 19.35 | 1.72E-05 | **5.85E-05** | 0.62 |
| lh_precuneus | 3.36 | 6.85E-02 | 1.05E-01 | 0.33 | 4.09 | 4.50E-02 | 8.50E-02 | 0.38 | 11.98 | 6.50E-04 | **1.23E-03** | 0.43 |
| lh_rostralanteriorcingulate | 2.10 | 1.49E-01 | 2.12E-01 | 0.26 | 0.45 | 5.03E-01 | 5.90E-01 | -0.11 | 3.89 | 5.00E-02 | 6.29E-02 | 0.29 |
| lh_rostralmiddlefrontal | 20.04 | 1.42E-05 | **9.67E-05** | 0.81 | 14.71 | 1.88E-04 | **1.60E-03** | 0.76 | 22.73 | 3.46E-06 | **1.96E-05** | 0.65 |
| lh_superiorfrontal | 7.88 | 5.60E-03 | **1.12E-02** | 0.49 | 7.50 | 6.97E-03 | **2.01E-02** | 0.51 | 15.37 | 1.19E-04 | **3.12E-04** | 0.51 |
| lh_superiorparietal | 0.38 | 5.40E-01 | 6.12E-01 | 0.10 | 1.48 | 2.26E-01 | 3.31E-01 | 0.21 | 15.09 | 1.37E-04 | **3.33E-04** | 0.49 |
| lh_superiortemporal | 4.59 | 3.36E-02 | 5.45E-02 | -0.31 | 5.54 | 1.99E-02 | **4.73E-02** | -0.41 | 12.96 | 3.96E-04 | **8.42E-04** | -0.49 |
| lh_supramarginal | 18.11 | 3.51E-05 | **1.71E-04** | 0.73 | 13.13 | 4.04E-04 | **2.29E-03** | 0.62 | 24.66 | 1.40E-06 | **1.19E-05** | 0.64 |
| lh_frontalpole | 1.49 | 2.24E-01 | 2.93E-01 | 0.20 | 0.06 | 8.09E-01 | 8.33E-01 | 0.04 | 1.25 | 2.65E-01 | 2.90E-01 | 0.15 |
| lh_temporalpole | 0.01 | 9.28E-01 | 9.28E-01 | 0.02 | 0.57 | 4.52E-01 | 5.49E-01 | -0.13 | 0.57 | 4.52E-01 | 4.66E-01 | 0.11 |
| lh_transversetemporal | 0.06 | 8.12E-01 | 8.39E-01 | 0.04 | 6.25 | 1.36E-02 | **3.55E-02** | -0.40 | 1.82 | 1.78E-01 | 2.09E-01 | -0.18 |
| lh_insula | 47.41 | 1.20E-10 | **4.09E-09** | 1.05 | 40.72 | 2.32E-09 | **7.90E-08** | 1.03 | 21.78 | 5.42E-06 | **2.63E-05** | 0.60 |

Table S11. Pearson’s *r* spatial correlations between expression of 22q11.2 genes and 22q11DS surface area deviance (ΔSA) severity by age subgroup, adjusted for age, sex, and scanner.

|  | Childhood (≤12 y) | | | Adolescence (13-17 y) | | | Adult (≥18 y) | | |
| --- | --- | --- | --- | --- | --- | --- | --- | --- | --- |
| Gene | Pearson *r* | Bootstrap Pearson *r* Mean (SD) | Pearson *r* Z-Score *P*_AHBA_ | Pearson *r* | Bootstrap Pearson *r* Mean (SD) | Pearson *r* Z-Score *P*_AHBA_ | Pearson *r* | Bootstrap Pearson *r* Mean (SD) | Pearson *r* Z-Score *P*_AHBA_ |
| **AIFM3** | **0.40** | **0.40 (0.13)** | **0.029** | 0.39 | 0.39 (0.13) | 0.062 | **0.42** | **0.41 (0.14)** | **0.034** |
| ARVCF | 0.11 | 0.10 (0.18) | 0.365 | -0.06 | -0.05 (0.17) | 0.545 | -0.04 | -0.04 (0.18) | 0.519 |
| C22orf39 | -0.36 | -0.35 (0.17) | 0.871 | -0.42 | -0.39 (0.16) | 0.927 | -0.40 | -0.4 (0.16) | 0.897 |
| CLDN5 | 0.25 | 0.25 (0.16) | 0.171 | 0.22 | 0.21 (0.16) | 0.245 | 0.29 | 0.29 (0.16) | 0.168 |
| COMT | 0.08 | 0.04 (0.2) | 0.436 | -0.05 | -0.08 (0.17) | 0.579 | -0.02 | -0.04 (0.18) | 0.512 |
| DGCR2 | 0.15 | 0.16 (0.17) | 0.295 | 0.25 | 0.25 (0.13) | 0.200 | 0.25 | 0.25 (0.14) | 0.168 |
| DGCR6 | -0.44 | -0.44 (0.12) | 0.983 | -0.29 | -0.29 (0.14) | 0.840 | -0.32 | -0.32 (0.13) | 0.886 |
| DGCR6L | -0.49 | -0.49 (0.12) | 0.994 | -0.44 | -0.43 (0.13) | 0.958 | -0.41 | -0.41 (0.14) | 0.932 |
| **DGCR8** | **0.47** | **0.46 (0.14)** | **0.014** | **0.53** | **0.52 (0.13)** | **0.005** | **0.54** | **0.52 (0.13)** | **0.006** |
| GNB1L | -0.10 | -0.11 (0.16) | 0.625 | -0.10 | -0.09 (0.15) | 0.595 | -0.02 | -0.02 (0.14) | 0.496 |
| GP1BB | -0.28 | -0.28 (0.16) | 0.842 | -0.19 | -0.18 (0.15) | 0.707 | -0.26 | -0.26 (0.13) | 0.838 |
| HIRA | -0.02 | -0.02 (0.17) | 0.505 | 0.10 | 0.08 (0.14) | 0.392 | 0.08 | 0.07 (0.16) | 0.399 |
| KLHL22 | -0.35 | -0.35 (0.14) | 0.924 | -0.29 | -0.27 (0.16) | 0.818 | -0.25 | -0.25 (0.16) | 0.749 |
| MED15 | -0.07 | -0.07 (0.19) | 0.558 | -0.02 | -0.01 (0.18) | 0.501 | -0.06 | -0.06 (0.18) | 0.535 |
| MRPL40 | -0.30 | -0.3 (0.17) | 0.836 | -0.32 | -0.31 (0.15) | 0.857 | -0.24 | -0.23 (0.16) | 0.724 |
| P2RX6 | 0.22 | 0.21 (0.16) | 0.225 | 0.20 | 0.19 (0.16) | 0.261 | 0.23 | 0.22 (0.16) | 0.22 |
| PI4KA | -0.24 | -0.25 (0.13) | 0.849 | -0.25 | -0.25 (0.14) | 0.786 | -0.26 | -0.26 (0.15) | 0.789 |
| PRODH | -0.07 | -0.06 (0.19) | 0.548 | -0.10 | -0.09 (0.18) | 0.596 | -0.15 | -0.14 (0.17) | 0.619 |
| RANBP1 | 0.11 | 0.1 (0.16) | 0.357 | 0.12 | 0.12 (0.12) | 0.347 | 0.15 | 0.15 (0.13) | 0.271 |
| RIMBP3 | -0.28 | -0.27 (0.16) | 0.821 | -0.35 | -0.34 (0.16) | 0.885 | -0.34 | -0.32 (0.19) | 0.769 |
| RTN4R | -0.28 | -0.29 (0.17) | 0.825 | -0.30 | -0.31 (0.13) | 0.855 | -0.33 | -0.34 (0.14) | 0.872 |
| SCARF2 | 0.35 | 0.35 (0.16) | 0.090 | 0.27 | 0.26 (0.15) | 0.188 | 0.27 | 0.27 (0.17) | 0.193 |
| SEPT5 | -0.40 | -0.41 (0.16) | 0.935 | -0.37 | -0.36 (0.15) | 0.908 | -0.37 | -0.36 (0.15) | 0.882 |
| SLC25A1 | -0.18 | -0.17 (0.2) | 0.660 | -0.11 | -0.12 (0.19) | 0.628 | -0.14 | -0.16 (0.16) | 0.648 |
| SLC7A4 | -0.46 | -0.45 (0.15) | 0.959 | -0.50 | -0.49 (0.14) | 0.983 | -0.52 | -0.51 (0.15) | 0.972 |
| SNAP29 | -0.43 | -0.43 (0.13) | 0.979 | -0.42 | -0.41 (0.14) | 0.940 | -0.49 | -0.47 (0.12) | 0.983 |
| TANGO2 | 0.18 | 0.18 (0.18) | 0.279 | 0.23 | 0.22 (0.17) | 0.227 | 0.24 | 0.23 (0.16) | 0.219 |
| UFD1 | 0.09 | 0.06 (0.18) | 0.407 | 0.00 | -0.01 (0.14) | 0.497 | 0.01 | 0 (0.13) | 0.47 |

Table S12. Pearson’s *r* spatial correlations between expression of 22q11.2 genes and 22q11DS AD cortical thickness deviance (ΔCT) severity by age subgroup, adjusted for age, age^2^, sex, and scanner.

|  | Childhood (≤12 y) | | | Adolescence (13-17 y) | | | Adult (≥18 y) | | |
| --- | --- | --- | --- | --- | --- | --- | --- | --- | --- |
| Gene | Pearson *r* | Bootstrap Pearson *r* Mean (SD) | Pearson *r* Z-Score *P*_AHBA_ | Pearson *r* | Bootstrap Pearson *r* Mean (SD) | Pearson *r* Z-Score *P*_AHBA_ | Pearson *r* | Bootstrap Pearson *r* Mean (SD) | Pearson *r* Z-Score *P*_AHBA_ |
| AIFM3 | 0.03 | 0.03 (0.18) | 0.409 | 0.27 | 0.27 (0.17) | 0.178 | 0.42 | 0.44 (0.16) | 0.104 |
| ARVCF | 0.06 | 0.06 (0.16) | 0.358 | 0.06 | 0.05 (0.16) | 0.423 | -0.11 | -0.07 (0.16) | 0.61 |
| C22orf39 | -0.14 | -0.13 (0.17) | 0.667 | -0.25 | -0.24 (0.18) | 0.797 | -0.29 | -0.35 (0.16) | 0.897 |
| CLDN5 | 0.07 | 0.07 (0.13) | 0.319 | 0.17 | 0.18 (0.15) | 0.248 | 0.37 | 0.25 (0.18) | 0.259 |
| COMT | 0.06 | 0.05 (0.13) | 0.356 | 0.06 | 0.06 (0.14) | 0.399 | -0.02 | -0.01 (0.17) | 0.529 |
| DGCR2 | 0.01 | 0.02 (0.17) | 0.434 | 0.14 | 0.14 (0.18) | 0.331 | 0.22 | 0.31 (0.17) | 0.205 |
| DGCR6 | -0.20 | -0.19 (0.16) | 0.767 | -0.18 | -0.17 (0.16) | 0.744 | -0.12 | -0.18 (0.13) | 0.769 |
| DGCR6L | -0.03 | -0.01 (0.18) | 0.477 | -0.07 | -0.07 (0.18) | 0.596 | -0.14 | -0.16 (0.16) | 0.714 |
| *DGCR8* | 0.09 | 0.08 (0.15) | 0.318 | 0.18 | 0.17 (0.17) | 0.290 | 0.39 | 0.37 (0.18) | 0.171 |
| **GNB1L** | 0.29 | 0.29 (0.15) | 0.073 | **0.34** | **0.34 (0.12)** | **0.044** | 0.34 | 0.31 (0.15) | 0.168 |
| GP1BB | -0.18 | -0.18 (0.18) | 0.725 | -0.17 | -0.17 (0.18) | 0.720 | -0.29 | -0.26 (0.15) | 0.818 |
| HIRA | -0.04 | -0.04 (0.15) | 0.533 | 0.05 | 0.05 (0.14) | 0.424 | 0.32 | 0.26 (0.14) | 0.202 |
| KLHL22 | 0.08 | 0.08 (0.2) | 0.352 | 0.10 | 0.11 (0.17) | 0.355 | 0.27 | 0.09 (0.2) | 0.439 |
| MED15 | 0.18 | 0.17 (0.17) | 0.212 | 0.13 | 0.12 (0.17) | 0.350 | -0.15 | 0.12 (0.15) | 0.382 |
| MRPL40 | 0.10 | 0.11 (0.12) | 0.238 | 0.16 | 0.17 (0.14) | 0.242 | 0.37 | 0.22 (0.16) | 0.266 |
| **P2RX6** | 0.28 | 0.27 (0.14) | 0.078 | **0.41** | **0.4 (0.14)** | **0.049** | **0.53** | **0.51 (0.12)** | **0.012** |
| PI4KA | 0.04 | 0.03 (0.16) | 0.404 | 0.09 | 0.08 (0.17) | 0.396 | -0.09 | -0.12 (0.15) | 0.669 |
| PRODH | -0.20 | -0.19 (0.15) | 0.785 | -0.23 | -0.23 (0.16) | 0.812 | -0.28 | -0.31 (0.17) | 0.829 |
| RANBP1 | 0.17 | 0.18 (0.15) | 0.190 | 0.17 | 0.16 (0.14) | 0.261 | 0.32 | 0.3 (0.12) | 0.114 |
| RIMBP3 | 0.17 | 0.18 (0.16) | 0.187 | -0.01 | 0.01 (0.18) | 0.499 | -0.08 | -0.14 (0.2) | 0.658 |
| RTN4R | 0.24 | 0.25 (0.17) | 0.137 | 0.07 | 0.07 (0.2) | 0.413 | -0.15 | -0.15 (0.2) | 0.66 |
| SCARF2 | 0.16 | 0.14 (0.15) | 0.233 | 0.24 | 0.24 (0.18) | 0.211 | 0.33 | 0.26 (0.17) | 0.248 |
| SEPT5 | -0.09 | -0.08 (0.17) | 0.593 | -0.08 | -0.08 (0.19) | 0.609 | -0.17 | -0.22 (0.16) | 0.775 |
| SLC25A1 | -0.35 | -0.34 (0.16) | 0.913 | -0.33 | -0.32 (0.16) | 0.892 | -0.25 | -0.33 (0.15) | 0.881 |
| SLC7A4 | -0.08 | -0.08 (0.16) | 0.596 | -0.26 | -0.24 (0.17) | 0.811 | -0.47 | -0.49 (0.14) | 0.98 |
| SNAP29 | -0.19 | -0.19 (0.13) | 0.828 | -0.29 | -0.29 (0.12) | 0.935 | -0.38 | -0.44 (0.13) | 0.978 |
| TANGO2 | 0.12 | 0.12 (0.18) | 0.284 | 0.18 | 0.18 (0.17) | 0.269 | 0.39 | 0.37 (0.15) | 0.12 |
| UFD1 | 0.00 | 0.01 (0.17) | 0.436 | 0.02 | 0.02 (0.17) | 0.475 | 0.08 | 0.01 (0.16) | 0.507 |

Table S13. 22q11.2 gene PLS1 loadings for surface area deviance (ΔSA) model.

| ΔSA PLS1 Loading Rank | Gene | Bootstrap Mean Loading Weight (SD) | Bootstrap Loading Weight Z-Score | Bootstrap Loading Weight Z-Score AHBA Rank | Bootstrap Loading Weight Z-Score *P*_AHBA_ |
| --- | --- | --- | --- | --- | --- |
| **1** | **DGCR8** | **0.0138 (0.0023)** | **5.8986** | **0.9983** | **0.0017** |
| **2** | **AIFM3** | **0.0167 (0.005)** | **3.3301** | **0.9565** | **0.0435** |
| 3 | SCARF2 | 0.0062 (0.0034) | 1.8487 | 0.8409 | 0.1591 |
| 4 | CLDN5 | 0.0061 (0.0035) | 1.7427 | 0.8290 | 0.1710 |
| 5 | DGCR2 | 0.0046 (0.003) | 1.5740 | 0.8064 | 0.1936 |
| 6 | P2RX6 | 0.0142 (0.0103) | 1.3831 | 0.7781 | 0.2219 |
| 7 | TANGO2 | 0.0053 (0.0042) | 1.2422 | 0.7573 | 0.2427 |
| 8 | RANBP1 | 0.0023 (0.0026) | 0.8670 | 0.6929 | 0.3071 |
| 9 | UFD1 | 0.0008 (0.0035) | 0.2446 | 0.5712 | 0.4288 |
| 10 | ARVCF | 0.0009 (0.0075) | 0.1262 | 0.5467 | 0.4533 |
| 11 | HIRA | 0.0004 (0.004) | 0.1076 | 0.5439 | 0.4561 |
| 12 | COMT | 0.0005 (0.0047) | 0.0983 | 0.5417 | 0.4583 |
| 13 | MED15 | -0.0007 (0.0022) | -0.3165 | 0.4566 | 0.5434 |
| 14 | PRODH | -0.0043 (0.0113) | -0.3781 | 0.4446 | 0.5554 |
| 15 | GNB1L | -0.0014 (0.003) | -0.4686 | 0.4270 | 0.5730 |
| 16 | SLC25A1 | -0.0031 (0.0042) | -0.7447 | 0.3704 | 0.6296 |
| 17 | MRPL40 | -0.0039 (0.0026) | -1.4997 | 0.2340 | 0.7660 |
| 18 | RIMBP3 | -0.0099 (0.0057) | -1.7253 | 0.2008 | 0.7992 |
| 19 | PI4KA | -0.0051 (0.0029) | -1.7364 | 0.1987 | 0.8013 |
| 20 | KLHL22 | -0.0053 (0.003) | -1.7545 | 0.1967 | 0.8033 |
| 21 | GP1BB | -0.0082 (0.0042) | -1.9298 | 0.1732 | 0.8268 |
| 22 | RTN4R | -0.008 (0.004) | -2.0103 | 0.1628 | 0.8372 |
| 23 | DGCR6 | -0.0068 (0.0031) | -2.1921 | 0.1418 | 0.8582 |
| 24 | SEPT5 | -0.0114 (0.0039) | -2.9506 | 0.0788 | 0.9212 |
| 25 | C22orf39 | -0.0094 (0.003) | -3.1750 | 0.0646 | 0.9354 |
| 26 | DGCR6L | -0.0079 (0.0021) | -3.7103 | 0.0391 | 0.9609 |
| 27 | SNAP29 | -0.0095 (0.0018) | -5.2629 | 0.0074 | 0.9926 |
| 28 | SLC7A4 | -0.0199 (0.0035) | -5.6512 | 0.0044 | 0.9956 |

Table S14. 22q11.2 gene PLS1 loadings for cortical thickness deviance (ΔCT) model.

| ΔCT PLS1 Loading Rank | Gene | Bootstrap Mean Loading Weight (SD) | Bootstrap Loading Weight Z-Score | Bootstrap Loading Weight Z-Score AHBA Rank | Bootstrap Loading Weight Z-Score *P*_AHBA_ |
| --- | --- | --- | --- | --- | --- |
| **1** | **P2RX6** | **0.0332 (0.0002)** | **4.6932** | **0.9933** | **0.0067** |
| **2** | GNB1L | 0.0071 (0.0001) | 2.1180 | 0.8792 | 0.1208 |
| 3 | TANGO2 | 0.0068 (0.0001) | 1.6862 | 0.8315 | 0.1685 |
| 4 | AIFM3 | 0.0115 (0.0002) | 1.6488 | 0.8272 | 0.1728 |
| 5 | *DGCR8* | 0.0061 (0.0001) | 1.5251 | 0.8066 | 0.1934 |
| 6 | RANBP1 | 0.0041 (0.0001) | 1.4578 | 0.7963 | 0.2037 |
| 7 | SCARF2 | 0.005 (0.0001) | 1.3994 | 0.7874 | 0.2126 |
| 8 | CLDN5 | 0.0048 (0.0001) | 1.3078 | 0.7744 | 0.2256 |
| 9 | MRPL40 | 0.0025 (0.0001) | 1.1663 | 0.7498 | 0.2502 |
| 10 | MED15 | 0.0018 (0.0001) | 0.8498 | 0.6948 | 0.3052 |
| 11 | DGCR2 | 0.0034 (0.0001) | 0.8380 | 0.6920 | 0.3080 |
| 12 | HIRA | 0.0026 (0.0001) | 0.7343 | 0.6692 | 0.3308 |
| 13 | KLHL22 | 0.0019 (0.0001) | 0.4686 | 0.6167 | 0.3833 |
| 14 | COMT | 0.0016 (0.0001) | 0.3959 | 0.5994 | 0.4006 |
| 15 | RTN4R | 0.0022 (0.0002) | 0.3706 | 0.5932 | 0.4068 |
| 16 | ARVCF | 0.0012 (0.0002) | 0.1725 | 0.5440 | 0.4560 |
| 17 | RIMBP3 | 0.0005 (0.0002) | 0.0727 | 0.5182 | 0.4818 |
| 18 | UFD1 | 0.0002 (0.0001) | 0.0645 | 0.5154 | 0.4846 |
| 19 | PI4KA | 0 (0.0001) | -0.0052 | 0.4975 | 0.5025 |
| 20 | DGCR6L | -0.0013 (0.0001) | -0.4193 | 0.3977 | 0.6023 |
| 21 | SEPT5 | -0.0041 (0.0002) | -0.7666 | 0.3247 | 0.6753 |
| 22 | DGCR6 | -0.0037 (0.0001) | -1.2169 | 0.2396 | 0.7604 |
| 23 | GP1BB | -0.0079 (0.0002) | -1.3150 | 0.2233 | 0.7767 |
| 24 | PRODH | -0.0165 (0.0003) | -1.8444 | 0.1439 | 0.8561 |
| 25 | C22orf39 | -0.0068 (0.0001) | -1.8653 | 0.1411 | 0.8589 |
| 26 | SLC25A1 | -0.009 (0.0001) | -2.0659 | 0.1170 | 0.8830 |
| 27 | SLC7A4 | -0.0128 (0.0002) | -2.1730 | 0.1035 | 0.8965 |
| 28 | SNAP29 | -0.0074 (0.0001) | -2.9221 | 0.0452 | 0.9548 |

Table S15 (separate file). MicroRNAs (miRNAs) found to be significantly differentially expressed in prefrontal cortex (PFC) or hippocampus (HP) in 22q11DS mouse models, and accounted for by DGCR8 haploinsufficiency in Stark et al., (2008) or Earls et al., (2012). miRNAs are listed by study and brain region.

Table S16 (separate file). Unique experimentally validated gene targets of microRNAs (miRNAs) with any evidence type in miRTarBase v7.0 found to be significantly differentially expressed in prefrontal cortex (PFC) or hippocampus (HP) in 22q11DS mouse models, and accounted for by DGCR8 haploinsufficiency in Stark et al., (2008) or Earls et al., (2012). Differentially expressed miRNAs targeting each gene are listed per study and brain region source.

Table S17 (separate file). Unique experimentally validated gene targets of microRNAs (miRNAs) with strong experimental evidence in miRTarBase v7.0 found to be significantly differentially expressed in prefrontal cortex (PFC) or hippocampus (HP) in 22q11DS mouse models, and accounted for by DGCR8 haploinsufficiency in Stark et al., (2008) or Earls et al., (2012). Differentially expressed miRNAs targeting each gene are listed per study and brain region source.
